# CMTM6 shapes antitumor T cell response through modulating protein expression of CD58 and PD-L1

**DOI:** 10.1016/j.ccell.2023.08.008

**Published:** 2023-09-07

**Authors:** Beiping Miao, Zhaoqing Hu, Riccardo Mezzadra, Lotte Hoeijmakers, Astrid Fauster, Shangce Du, Zhi Yang, Melanie Sator-Schmitt, Helena Engel, Xueshen Li, Caroline Broderick, Guangzhi Jin, Raquel Gomez-Eerland, Lisette Rozeman, Xin Lei, Hitoshi Matsuo, Chen Yang, Ingrid Hofland, Dennis Peters, Annegien Broeks, Elke Laport, Annika Fitz, Xiyue Zhao, Mohamed A.A. Mahmoud, Xiujian Ma, Sandrine Sander, Hai-kun Liu, Guoliang Cui, Yu Gan, Wei Wu, Yanling Xiao, Albert J.R. Heck, Wenxian Guan, Scott W. Lowe, Hugo M. Horlings, Cun Wang, Thijn R. Brummelkamp, Christian U. Blank, Ton N.M. Schumacher, Chong Sun

**Affiliations:** 1 German Cancer Research Center (DKFZ) Heidelberg, Division Immune Regulation in Cancer, Im Neuenheimer Feld 280, 69120 Heidelberg, Germany; 2 State Key Laboratory of Oncogenes and Related Genes, Shanghai Cancer Institute, Renji Hospital, Shanghai Jiao Tong University School of Medicine, Shanghai, China; 3 Faculty of Biosciences, Heidelberg University, 69120 Heidelberg, Germany; 4 Division of Molecular Oncology & Immunology, Netherlands Cancer Institute, Oncode Institute, Plesmanlaan 121, 1066 CX Amsterdam, the Netherlands; 5 Department of Cancer Biology and Genetics, Memorial Sloan Kettering Cancer Center, New York, NY 10065, USA; 6 Division of Biochemistry, Netherlands Cancer Institute, Oncode Institute, Plesmanlaan 121, 1066 CX Amsterdam, the Netherlands; 7 Faculty of Medicine, Heidelberg University, 69120 Heidelberg, Germany; 8 Department of Gastrointestinal Surgery, Nanjing Drum Tower Hospital, The Affiliated Hospital of Nanjing University Medical School, Nanjing, China; 9 Department of Biomedical Engineering, Stevens Institute of Technology, Hoboken, NJ 07030, USA; 10 Department of Interventional Radiology, Tongren Hospital, Shanghai Jiao Tong University School of Medicine, No. 1111, Xianxia Road, Shanghai 200336, China; 11 Department of Immunology, Leiden University Medical Center (LUMC), Leiden, the Netherlands; 12 Core Facility Molecular Pathology & Biobanking, Netherlands Cancer Institute, Plesmanlaan 121, 1066 CX Amsterdam, the Netherlands; 13 German Cancer Research Center (DKFZ) Heidelberg, Division Molecular Neurogenetics, DKFZ-ZMBH Alliance, Im Neuenheimer Feld 280, 69120 Heidelberg, Germany; 14 German Cancer Research Center (DKFZ) Heidelberg, Division Adaptive Immunity and Lymphoma, Im Neuenheimer Feld 280, 69120 Heidelberg, Germany; 15 German Cancer Research Center (DKFZ) Heidelberg, Division T Cell Metabolism, Im Neuenheimer Feld 280, 69120 Heidelberg, Germany; 16 Biomolecular Mass Spectrometry and Proteomics, Bijvoet Center for Biomolecular Research and Utrecht Institute for Pharmaceutical Sciences, Padualaan 8, 3584 CH Utrecht, the Netherlands; 17 Netherlands Proteomics Centre, Padualaan 8, 3584 CH Utrecht, the Netherlands; 18 Singapore Immunology Network (SIgN), Agency for Science, Technology and Research (A*STAR), Singapore 138648, Singapore; 19 Department of Pharmacy, National University of Singapore, Singapore 117543, Singapore; 20 Department of Pathology, Netherlands Cancer Institute, Plesmanlaan 121, 1066 CX Amsterdam, the Netherlands; 21 Department of Medical Oncology, Netherlands Cancer Institute (NKI), Plesmanlaan 121, 1066 CX Amsterdam, the Netherlands; 22 Department of Medical Oncology, Leiden University Medical Centre (LUMC), Leiden, The Netherlands; 23 Department of Hematology, Leiden University Medical Center (LUMC), Leiden, the Netherlands; 24 These authors contributed equally; 25 Lead contact

## Abstract

The dysregulated expression of immune checkpoint molecules enables cancer cells to evade immune destruction. While blockade of inhibitory immune checkpoints like PD-L1 forms the basis of current cancer immunotherapies, a deficiency in costimulatory signals can render these therapies futile. CD58, a costimulatory ligand, plays a crucial role in antitumor immune responses, but the mechanisms controlling its expression remain unclear. Using two systematic approaches, we reveal that CMTM6 positively regulates CD58 expression. Notably, CMTM6 interacts with both CD58 and PD-L1, maintaining the expression of these two immune checkpoint ligands with opposing functions. Functionally, the presence of CMTM6 and CD58 on tumor cells significantly affects T cell-tumor interactions and response to PD-L1–PD-1 blockade. Collectively, these findings provide fundamental insights into CD58 regulation, uncover a shared regulator of stimulatory and inhibitory immune checkpoints, and highlight the importance of tumor-intrinsic CMTM6 and CD58 expression in antitumor immune responses.

## In brief

Miao et al. reveal CMTM6 as a positive regulator and interaction partner of CD58, an important costimulatory ligand in antitumor immune responses. They highlight the critical roles of tumor-intrinsic CMTM6 and CD58 expression in modulating T cell-tumor cell interactions and their potential impact on T cell-based immunotherapy.

## Graphical abstract

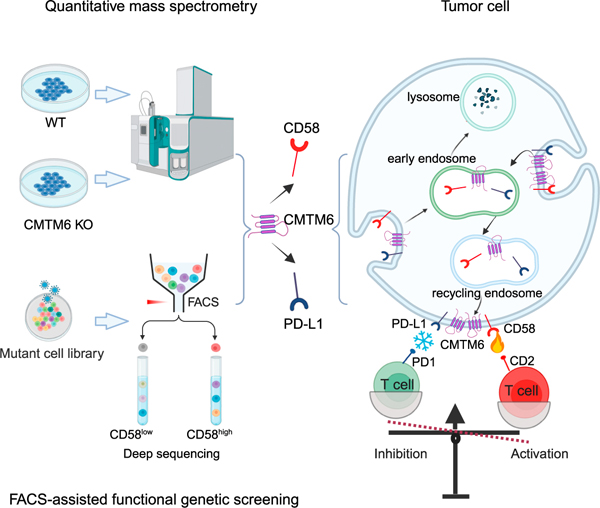

## INTRODUCTION

Tumor-reactive T cells form a major component of antitumor immunity and are under tight regulation by an array of coinhibitory and costimulatory immune checkpoints. Malignant tumors often exploit dysregulated expression of these checkpoint molecules to evade immune destruction.^1–4^ Clinical targeting of CTLA-4 and the PD-L1-PD-1 axis has revolutionized cancer treatment, leading to durable responses in some patients.^1–7^ However, primary and acquired resistance to these immune checkpoint blockade (ICB) therapies remains a major limitation. Recent studies indicate that upregulation of alternative coinhibitory immune checkpoint receptors or ligands within the tumor microenvironment (TME) is associated with the unfavorable responses.^8–14^ Alternatively, loss of costimulatory immune checkpoints has also been linked to immunotherapy resistance.^15–17^

CD58 is a glycoprotein expressed widely across human tissues, and its receptor CD2 is primarily found on T cells and natural killer (NK) cells.^18–21^ When CD58 engages with CD2, it promotes cell-cell adhesion and delivers a costimulatory signal to CD2-expressing cells.^22–26^ Abrogation of CD58−CD2 interaction by genetic deletion or blocking antibodies impairs the adhesion, activation, and cytolytic activities of T cells and NK cells, leading to compromised antitumor immune response in melanoma,^17,27^ B cell lymphoma,^28^ glioma,^29^ neuroblastoma,^30^ ovarian cancer,^31^ and gastric cancer models.^31^ Conversely, increased CD58 expression by virus-mediated ectopic gene expression in a colorectal cancer model^32^ or by an EZH2 inhibitor in B cell lymphoma cells^33^ potentiated antitumor immunity. In line with these data, mutations and loss of CD58 expression are frequently observed in human leukemia and lymphomas, serving as adverse prognostic factors.^28,30,34–38^ Notably, CD58 deficiency in large B cell lymphomas hampers their response to chimeric antigen receptor-T (CAR-T) cell therapy.^16,38^ Moreover, melanoma that has developed resistance to ICB treatment exhibits significantly reduced CD58 expression compared to treatment-naive tumors.^17^

Despite the critical role of CD58 in antitumor immunity, the mechanisms that control its expression have been poorly understood. In this study, we found that CMTM6 (CKLF like MARVEL transmembrane domain containing 6) plays a key role in promoting the expression of both CD58 and PD-L1, two immune checkpoint ligands with opposing functions, in tumor cells. Additionally, we elucidated the underlying mechanisms, functional consequences, and clinical implications of this regulation in the antitumor T cell response.

## RESULTS

### CMTM6 is a positive regulator of CD58

**Figure 1.**
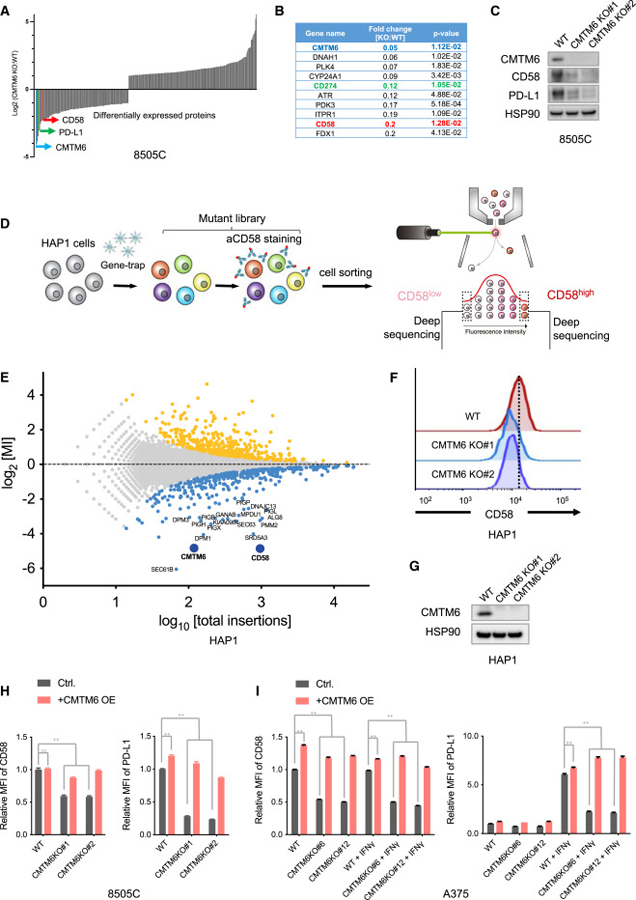
Identification of CMTM6 as a positive modulator of CD58 (A) Quantitative proteomics analysis of CMTM6-proficient (WT) and -deficient (KO) 8505C cells. Fold changes of significantly differentially expressed proteins (two-sided Student’s *t* test, p < 0.05) are depicted. (B) List of top 10 downregulated proteins in CMTM6-deficient 8505C cells. (C) Western blot analysis of CMTM6, CD58, and PD-L1 expression in parental 8505C cells (WT) and independent CMTM6-knockout clonal cells (CMTM6 KO). HSP90 served as a control. (D) Schematic illustration of the flow cytometry-based haploid genetic screen for modulators of CD58 expression. (E) Identification of modulators of CD58 expression by the haploid genetic screen depicted in (D). Each dot represents an individual gene, with the x axis indicating the number of disruptive insertions in each gene, and the y axis showing the fold changes in the frequency of unique insertions in the CD58^high^ population compared to the CD58^low^ population. Genes with a significant enrichment of insertions (two-sided Fisher’s exact test, FDR-corrected p < 0.05) in either the CD58^high^ or CD58^low^ populations are represented by light orange and blue dots, respectively. (F and G) CD58 expression levels in parental HAP1 cells (WT) and independent CMTM6-knockout clonal cells (CMTM6 KO). Levels of CD58 expression were determined by flow cytometry (F) and CMTM6 expression was analyzed by Western blot (G). HSP90 served as a control in the Western blot analysis. (H and I) Flow cytometry analysis of CD58 and PD-L1 expression in wild-type (WT), CMTM6-knockout (CMTM6 KO), CMTM6-overexpressing (WT + CMTM6 OE), and CMTM6-reconstituted (CMTM6 KO + CMTM6 OE) 8505C (H) and A375 cells with or without IFNγ exposure (I). Data represent the mean ± standard deviation of triplicates and were analyzed using a two-way ANOVA test (with Tukey’s multiple comparisons test). A p value greater than 0.05 indicates non-significance (ns), while a p value less than 0.0001 is denoted as **.

Previously, we and others unveiled CMTM6 as a positive regulator and molecular partner of PD-L1,^39,40^ marking the first recognized function of CMTM6. However, our understanding of CMTM6 remains limited. We quantitatively analyzed the total proteome of CMTM6-deficient and -proficient tumor cells. In addition to reduced PD-L1 expression, a significant downregulation of CD58 (LFA-3) was also observed in CMTM6-deficient cells (Figures 1A and 1B), which was further confirmed by Western blot analysis (Figure 1C).

Given the critical role of CD58 in antitumor immune responses, we conducted a FACS-based haploid genetic screen to systematically identify modulators of this protein (Figure 1D). As expected, disruptive insertions in the CD58 locus were highly enriched in the CD58^low^ cell population, underpinning the reliability of the genetic screen. Moreover, components of the glycosylphosphatidylinositol (GPI) biosynthesis pathway (e.g., DPM1, SRD5A3, PIGX, PIGH, etc) and the endoplasmic reticulum translocon complex (SEC61B, SEC63) were identified as regulators of CD58 expression, consistent with the fact that CD58 is expressed in both GPI-anchored and transmembrane forms. Notably, CMTM6 emerged as one of the most significant hits (Figure 1E). To verify this finding, we generated independent CMTM6-knockout clones in HAP1 cells and assessed the levels of CD58 expression. Compared to parental HAP1 cells, CMTM6-deficient cells showed reduced cell surface expression of CD58 (Figures 1F and 1G). Additionally, reintroducing CMTM6 expression in the CMTM6-deficient cells restored CD58 expression (Figures S1A and S1B).

### CMTM6 co-regulates CD58 and PD-L1 in tumor cells

The above comparative proteomic analysis (Figures 1A–1C) and the haploid genetic screen (Figures 1E–1G) both revealed CMTM6 as a positive regulator of the CD58 immune checkpoint. As CMTM6 has previously been shown to maintain PD-L1 protein expression,^39,40^ the results together present an intriguing case in which the expression of a costimulatory and a coinhibitory immune checkpoint ligands are controlled by the same regulatory protein.

To validate the regulatory role of CMTM6 in CD58 and PD-L1 expression, we conducted CMTM6 deletion and reconstitution in cell lines derived from multiple cancer types, including 8505C thyroid cancer cells (Figures 1H and S1C), A375 melanoma cells (Figures 1I and S1D), and RKO colorectal cancer cells (Figures S1E and S1F). In A375 cells, PD-L1 expression is induced by IFNγ, while CD58 expression is constitutive and not affected by the cytokine exposure. Disruption of CMTM6 by CRISPR-Cas9 diminished cell surface and total protein levels of CD58 and PD-L1. Conversely, reconstitution of CMTM6 restored the expression of both immune checkpoint ligands (Figures 1I and S1D). In 8505C and RKO cells that constitutively express high basal levels of CD58 and PD-L1, CMTM6 deletion concurrently downregulated both immune checkpoint ligands, while reintroducing CMTM6 reverted this phenotype (Figures 1H, S1C, S1E, and S1F). In addition to the solid tumor-derived cell models, we also observed reduced CD58 expression upon CMTM6 knockout in B cell lymphoma cells (BJAB and Ramos) and acute myeloid leukemia cells (OCI-AML2) (Figures S1G–S1I). Furthermore, CD58 is expressed on professional antigen-presenting cells. To assess the influence of CMTM6 on CD58 regulation in primary human dendritic cells (DCs), we generated DCs from human peripheral blood-derived progenitors^41^ and used RNAi to suppress CMTM6 expression in the cells. The results showed that CMTM6 suppression led to decreased CD58 levels in primary DCs (Figure S1J). To investigate whether CMTM6 only influences CD58 expression levels or also impacts its interaction with CD2, we identified a CD58-targeting short hairpin RNA (shRNA) that reduces CD58 expression to a level comparable to that observed in CMTM6-knockout cells (Figure S1K). Comparison of cells with matched CD58 expression levels generated by either CD58 knockdown or CMTM6 deficiency revealed comparable CD2 binding (Figure S1L), suggesting that CMTM6 does not impact CD2 binding capacity beyond its effect on CD58 expression levels.

Collectively, these results establish CMTM6 as a regulator of CD58 protein levels in various cancer types and in primary human DCs.

### CMTM6 maintains CD58 protein stability

**Figure 2.**
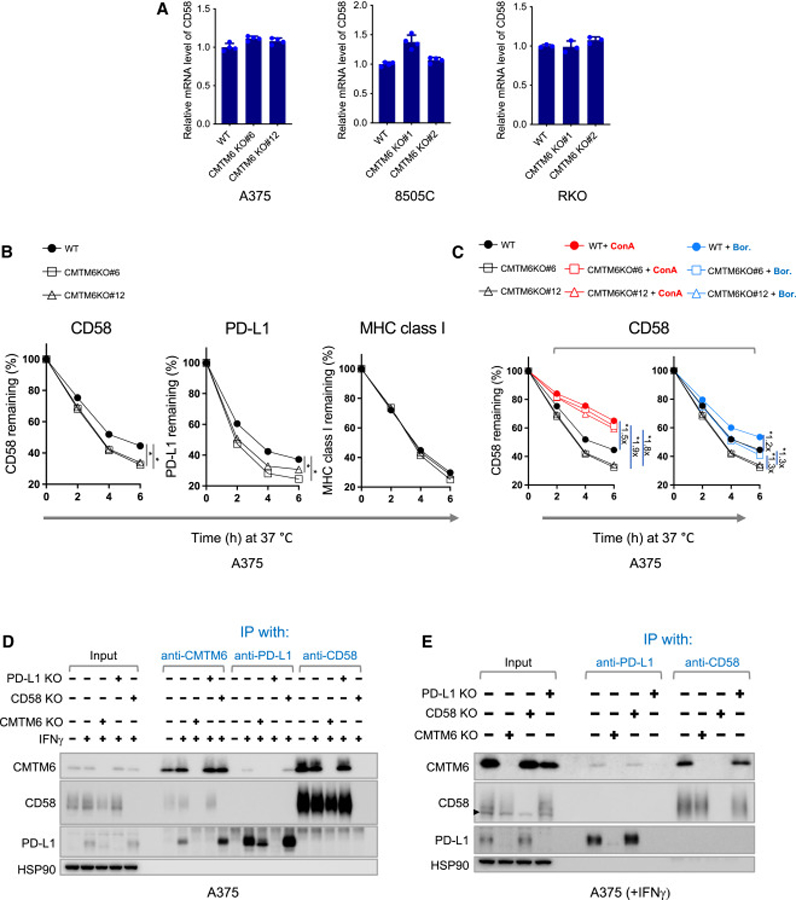
CMTM6 promotes stability of cell surface CD58 and interacts with CD58 (A) qPCR analysis of mRNA levels of *CD58* in CMTM6-deficient and -proficient A375, 8505C, and RKO cells. (B) Stability of cell surface-expressed CD58, PD-L1, and MHC class I in parental (WT) and CMTM6-deficient (CMTM6 KO#6, CMTM6 KO#12) A375 cells. A375 cells were treated with IFNγ for 24 h and then individually incubated with APC-conjugated antibodies specific for CD58, PD-L1, or MHC class I at 4°C. After removing unbound antibodies, the cells were further incubated at 37°C for the indicated time periods, and the APC signal was measured by flow cytometry. The percentage of signal remaining at the indicated time points relative to time 0 is shown. (C) Stability of cell surface-expressed CD58 in the parental (WT) and CMTM6-deficient (CMTM6 KO#6, CMTM6 KO#12) A375 cells in the presence of the proteasome inhibitor bortezomib (Bor.) or the lysosome inhibitor concanamycin A (ConA). The untreated samples presented in (B) served as the control. Data acquisition and presentation were performed as described in (B). (D) Western blot analysis of cell lysates and indicated immunoprecipitates from A375 cells. HSP90 served as a control. (E) Western blot analysis of cell lysates and indicated immunoprecipitates by cell surface immunoprecipitation from A375 cells. For the cell surface immunoprecipitation, live cells were incubated with antibodies that recognize the extracellular domains of CD58 or PD-L1. After removal of unbound antibodies, the cells were lysed for (co)immunoprecipitation. HSP90 served as a control. The triangles indicate the position of background bands, which are present when the anti-CD58 antibody (R&D Cat# AF1689) was used for detection, whereas the anti-CD58 antibody (BioLegend, Cat# 330924) does not produce such background signal. Data represent mean ± standard deviation of at least triplicates (A–C) and were analyzed using unpaired Student’s *t* test. Statistical significance is indicated by *p < 0.05.

To understand the mechanism of CMTM6-mediated CD58 regulation, we first compared CD58 mRNA levels in CMTM6-deficient and -proficient cells. CMTM6 depletion does not decrease the level of RNA transcripts of *CD58* (Figure 2A), while significantly reducing the cell surface and total CD58 protein levels (Figure 1), indicating the regulation occurs at a posttranscriptional level.

CMTM6 contains a tetra-spanning MARVEL (MAL and related proteins for vesicle trafficking and membrane link) domain.^42,43^ It promotes the endocytic recycling of PD-L1^40^ and protects PD-L1 from ubiquitination.^39^ We speculated that CMTM6 could regulate CD58 expression through a similar mechanism. To investigate this, we traced the fate of cell surface-expressed CD58 by labeling them with allophycocyanin (APC)-conjugated CD58-specific antibodies, and then quantified the APC signal over time. The analysis revealed a more rapid decay of cell surface CD58 in the absence of CMTM6 (Figure 2B). Consistent with previous reports,^39,40^ CMTM6 deficiency accelerated the degradation of PD-L1 but not major histocompatibility complex class I. In eukaryotic cells, protein degradation primarily occurs through the proteasome and lysosomal proteolysis pathways. To investigate the mechanisms involved in CMTM6-mediated CD58 regulation, we incubated cells with inhibitors of the proteasome or lysosome acidification and assessed the stability of the APC-labeled CD58. Both inhibitors delayed decay of the fluorescent signal. Notably, lysosome inhibition substantially reduced the rate of CD58 degradation in CMTM6-deficient cells to a level similar to that observed in CMTM6-proficient cells (Figure 2C). Proteasome inhibition led to a slightly greater increase in fluorescent signal in CMTM6-deficient cells than in CMTM6-proficient cells (Figure 2C). These results suggest that CMTM6 protects cell surface CD58 from lysosome-mediated degradation and, to a lesser extent, from proteasomal proteolysis. Consistently, immunofluorescence imaging revealed a significant overlap between the signals of CD58 and CMTM6 on the cell membrane. Moreover, both CD58 and CMTM6 exhibited partial colocalization with EEA1 (an early endosome marker) and LAMP1 (a late endosome/lysosome marker), while demonstrating a higher degree of colocalization with TFRC (a recycling endosome marker) (Figures S2A–S2D).

### CMTM6 interacts with CD58

As CMTM6 colocalizes with and regulates the stability of both PD-L1^39,40^ and CD58 (Figures S2, 2B, and 2C), we next assessed possible interactions between the three molecules. To this purpose, we performed co-immunoprecipitation experiments using anti-CMTM6, anti-CD58, and anti-PD-L1 antibodies, followed by immunoblot analysis. In anti-CMTM6 immunoprecipitates of A375 whole-cell lysates, both CD58 and PD-L1 were detected. Likewise, CMTM6 was present in both anti-CD58 and anti-PD-L1 immunoprecipitates (Figure 2D). However, PD-L1 was not detected in anti-CD58 immunoprecipitates and vice versa. Therefore, the data do not suggest there are direct interactions between CD58 and PD-L1 (Figure 2D). To determine whether CMTM6 interacts with cell surface PD-L1 and CD58 molecules, cell surface immunoprecipitation experiments were performed. In line with the prior data, molecular associations of CMTM6/CD58 and CMTM6/PD-L1 were individually observed, while no evidence for an interaction between CD58 and PD-L1 in the same molecular complex was obtained (Figure 2E). As a control for antibody specificity, lysates from cells that carry genetic inactivation of the *CMTM6*, *CD58*, or *PD-L1* genes were included, in which co-immunoprecipitation of the different partner molecules was no longer observed (Figures 2D and 2E). Interestingly, PD-L1 levels were slightly elevated in CD58-deficient A375 cells, consistent with the observation reported by Frangieh et al.^17^ Along with the elevated PD-L1 level, the association between PD-L1 and CMTM6 was increased, in line with the model that CMTM6 binds to PD-L1 and stabilizes it. In contrast, PD-L1 loss in tumor cells did not result in an obvious increase in the levels of CD58 or CMTM6/CD58 interactions (Figures 2D and 2E).

### CMTM6 loss in tumor cells compromises T cell activation

**Figure 3.**
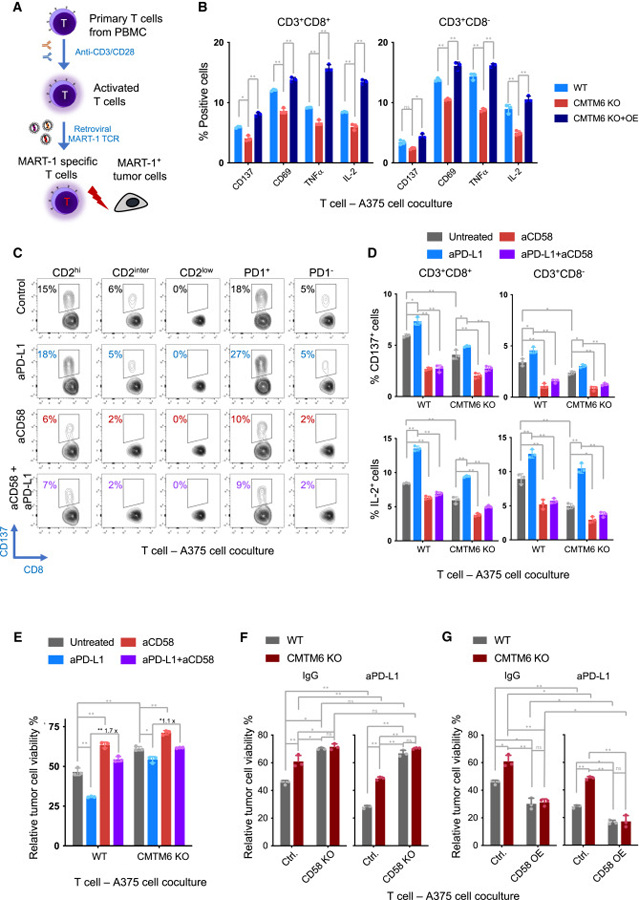
Influence of CMTM6, CD58, and PD-L1 on antigen-specific T cell-tumor cell interactions (A) Schematic illustration of the antigen-specific T cell-tumor cell coculture system. Primary T cells were isolated from human PBMCs, activated, and transduced with a MART-1-specific TCR. HLA-A2-positive tumor cells were loaded with MART-1 peptide and incubated with the TCR-transduced T cells. (B) Effect of CMTM6 loss on T cell activation. MART-1 TCR-transduced T cells were cocultured with MART-1 peptide-loaded CMTM6 wild-type (WT), CMTM6-knockout (CMTM6 KO), or CMTM6-reconstituted (CMTM6 KO + OE) A375 cells. After 24 h, expression of indicated T cell activation markers and cytokines in CD8^+^ and CD8^−^ T cells was determined by flow cytometry. To allow detection of TNFα and IL-2, brefeldin A was added 4 h before T cell harvesting. (C–E) Effect of PD-L1 blockade, CD58 blockade, and CMTM6 deletion on T cell activation and tumor cell viability. MART-1 TCR-transduced T cells were co-cultured with MART-1 peptide-loaded tumor cells in the presence of PD-L1-blocking antibody (atezolizumab, aPD-L1), CD58-blocking antibody (aCD58), or their combination. The coculture without antibody treatment (untreated) served as control. After 18–60 h of coculture, T cell activation and tumor cell viability were analyzed. (C) Flow cytometric analysis of T cells that were cocultured for 18 h with A375 cells in the absence or presence of the indicated blocking antibodies. Expression of CD137 was analyzed within CD3^+^CD8^+^ cell populations stratified by CD2 expression (CD2^high^: top 33%, CD2^inter^: middle 33%, CD2^low^: bottom 33%), as well as PD-1 expression (PD-1^+^ or PD-1^−^). Representative contour plots are presented. (D) Flow cytometric analysis of T cells that were cocultured with CMTM6 wild-type and -knockout A375 cells for 24 h. Percentages of CD137^+^, and IL-2^+^ cells within the CD8^+^CD3^+^ and CD8^−^ CD3^+^ cell populations are depicted. (E) Viability of CMTM6 wild-type and -knockout A375 cells after coculture with T cells for 60 h was determined by CellTiter-Blue Cell Viability Assay. The data presented depict relative tumor cell viability (relative to tumor cells that were cultured in the absence of T cells). (F and G) Viability of A375 cells, genetically modified as indicated, was assessed using the CellTiter-Blue Cell Viability Assay after 60 h of coculture with T cells. A375 cells with wild-type CMTM6 (Ctrl.) or CMTM6 knockout (CMTM6 KO), with or without additional CD58 knockout (CD58 KO) (F), or CD58 overexpression (CD58 OE) (G), were examined separately. Cells were either treated with a PD-L1-blocking antibody or a control IgG antibody, as specified. The data presented depict tumor cell viability relative to tumor cells that were cultured in the absence of T cells. Data represent mean ± standard deviation of at least triplicates (B) (D–G) and were analyzed using two-way ANOVA (Tukey’s multiple comparisons test). The statistical significance levels are indicated as follows: ns (not significant; p ≥ 0.05), * (p < 0.05), and ** (p < 0.0001).

We proceeded to evaluate the effects of CMTM6 in tumor cells on T cell activation. To recapitulate interactions between human tumor cells and tumor-reactive T cells, we cocultured melanoma antigen recognized by T cell 1 (MART-1) antigen-loaded tumor cells and T cells that were transduced with a MART-1-specific T cell receptor (Figure 3A). Following tumor recognition, T cells showed increased expression of activation markers (CD137, CD69, IL-2, and TNF-α) (Figure S3A). Moreover, PD-L1 blockade further enhanced T cell activation, while CD58 blockade had the opposite effect (Figure S3A). Notably, CD2^high^ T cells demonstrated pronounced increases in activation marker expression upon encountering tumor cells, whereas T cell activation levels were lower in CD2^inter^ T cells and became minimal in CD2^low^ T cells (Figures 3C and S3C), aligning with the critical role of the CD58 -CD2 pathway in T cell activation.^17,27,29–31^

Using this coculture model, we found that incubation with CMTM6-knockout tumor cells significantly reduced the expression of T cell activation markers (CD137 and CD69) and T cell effector cytokines (IL-2 and TNFα) compared to wild-type and CMTM6-reconstituted tumor cells (Figures 3B and S3B). Consistently, CMTM6 loss resulted in a notable increase in tumor cell viability (Figures 3E and S3G)

### Critical role of CD58 in T cell-tumor interactions and response to PD-L1 blockade

Given the dual roles of CMTM6 in maintaining the cell surface expression of both CD58 and PD-L1, we sought to determine the relative importance of these two immune checkpoints with opposing functions in T cell-tumor cell interactions. Remarkably, when CD58 and PD-L1 were simultaneously blocked, the enhanced T cell activation observed with PD-L1 blockade alone was completely abolished, both within CD2^high^ and the PD-1^+^ T cell populations (Figures 3C and S3C). Actually, co-inhibition of PD-L1 and CD58 reduced activation of CD2^high^ and CD2^inter^ T cells to a level lower than that of the untreated control, indicating a dominant effect of CD58 blockade. This significant impact of CD58 blockade on T cell activation was consistently observed in coculture systems using A375 and 8505C cells (Figures 3C and S3C), where PD-L1 and CD58 expression was detected, and antibody-based blockade was effective (Figure S3D).

The aforementioned data suggest a crucial role of CD58 in antigen-specific T cell-tumor cell interactions and the response to PD-L1 blockade. The compromised T cell activation observed upon CMTM6 loss in tumor cells (Figures 3B and S3B) may be attributed to the reduced expression of CD58. To test this hypothesis, we cocultured wild-type and CMTM6-knockout tumor cells with tumor-specific T cells in the presence of PD-L1- or CD58-blocking antibodies, or their combination. Consistently, impaired T cell activation was observed in the presence of CMTM6-deficient cells compared to wild-type cells (Figures 3D, S3E, and S3F). CD58 blockade by antibody further reduced T cell activation, while PD-L1 inhibition exerted the opposite effect (Figures 3D, S3E, and S3F). Additionally, reduction of CD58 expression by shRNA to the same level as observed in CMTM6-knockout cells resulted in a slightly more pronounced attenuation of T cell activation and improved the tumor cell survival compared to that observed in CMTM6-knockout cells. This difference in T cell activation was eliminated upon PD-L1 blockade, indicating that reduced PD-L1 expression in CMTM6-knockout cells is responsible for the observed difference (Figures S3H–S3I). These results indicate that the residual CD58 and PD-L1 at the surface of CMTM6-deficient tumor cells actively modulate T cell responses (Figures 3D, S3E, and S3F).

Remarkably, the combined blockade of CD58 and PD-L1 by antibodies largely offset the impact of PD-L1 blockade alone on T cell activation in both CMTM6-proficient and -deficient conditions (Figures 3D, S3E, and S3F), supporting the crucial role of CD58 in response to PD-L1 blockade. To further examine this, we generated CMTM6-proficient and -deficient tumor cells that were either CD58 knockout or overexpressing CD58 (Figure S4A). Co-culturing these cells with T cells demonstrated that CD58 knockout significantly reduced T cell activation (Figure S4B), whereas CD58 overexpression increased T cell activation (Figure S4C). Importantly, in both scenarios, manipulating CD58 levels through knockout and overexpression eliminated the difference in T cell activation between cocultures with either CMTM6-proficient or CMTM6-deficient tumor cells. Moreover, the effect of anti-PD-L1 antibodies on T cell activation was significantly attenuated in the absence of CMTM6 or CD58 (Figures S4B and S4C).

Consistent with the compromised T cell activation, we observed that loss of CMTM6 in A375 or 8505C cells resulted in increased tumor cell survival (Figures 3E and S3G). Furthermore, PD-L1 inhibition enhanced the killing of tumor cells by T cells, while, on top of that, inhibition of CD58 significantly rescued the tumor cells (Figures 3E and S3G). This rescue effect by CD58 inhibition was more pronounced in CMTM6-proficient cells compared to CMTM6-deficient cells (1.7-folds vs. 1.1-folds in A375, Figure 3E; 1.6-folds vs. 1.2-folds in 8505C; Figure S3G), potentially due to the lower level of CD58 in CMTM6-deficient cells. Supporting this, CD58 knockout abolished the differential response to tumor-specific T cells and PD-L1 blockade between CMTM6-proficient and -deficient cells (Figure 3F). Conversely, when CD58 was overexpressed in both CMTM6-deficient and CMTM6-proficient tumor cells, their viability decreased to a comparable level after coculture with tumor-reactive T cells (Figure 3G). These findings underscore the critical involvement of CD58 in antigen-specific T cell-tumor cell interactions and response to PD-L1 blockade, with CMTM6-mediated CD58 regulation playing a significant role in this context.

As of now, a murine homolog of CD58 has not been identified. To investigate the roles of CMTM6 and CD58 in the antitumor T cell response *in vivo*, we used an *in vivo* xenograft model with acute myeloid leukemia (AML) cells treated with CAR-T cells. In this model, a pool of CMTM6-proficient and -deficient AML cells were subjected to CAR-T treatment, and we observed a preferential survival of CMTM6-deficient tumor cells. As the dose of CAR-T cells increased, there was a higher enrichment of CMTM6-deficient cells. These results indicate that CMTM6 deficiency offers protection to tumor cells from CAR-T therapy. Additionally, when CD58 was inhibited, the enrichment of CMTM6-deficient tumor cells by CAR-T treatment was diminished (Figures S4D and S4E). These *in vivo* findings align with the *in vitro* data and suggest that the expression of CMTM6 in tumor cells contributes to the response to T cell-based immunotherapy by modulating CD58 expression levels.

### CMTM6 and CD58 expression in tumor cells is positively correlated and associated with clinical response to ICB therapies

**Figure 4.**
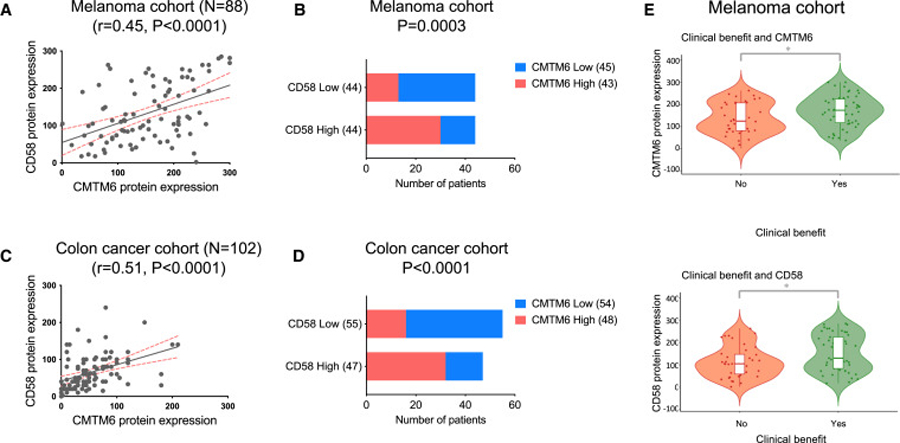
Correlation between CMTM6 and CD58 expression in tumor cells and their association with response to ICB therapies (A–D) Tumor biopsies from 88 melanoma and 102 colon cancer patients were analyzed for CMTM6 and CD58 expression levels using immunohistochemistry. (A, C) Spearman correlation analysis was performed to evaluate the correlation between CMTM6 and CD58 expression levels in tumor cells. (B and D) The association between CMTM6 and CD58 expression levels in tumor cells was analyzed using chi-squared tests. The samples were divided into CMTM6-high, CMTM6-low, CD58-high, and CD58-low groups based on the median expression values, and the results were presented as contingency tables. (E) The association between CMTM6 and CD58 expression in tumor cells and the response to ICB therapies in the melanoma cohort. The expression levels of CMTM6 and CD58 were individually plotted against the patient’s response to therapy, classified as clinical benefit (Yes) or no clinical benefit (No). Patients with complete response (CR), partial response (PR), and stable disease (SD) lasting for 6 months or more were classified as having clinical benefit (Yes, n = 49), while patients with progressive disease or with an SD for less than 6 months were categorized as having no clinical benefit (No, n = 39). Comparisons were made between patients that showed clinical benefit (Yes) and those that showed no clinical benefit (No) using the Wilcoxon rank-sum test. Statistical significance is indicated by *p < 0.05.

To investigate the relationship between CMTM6 and CD58 expression in human cancers, we conducted immunohistochemistry (IHC) analysis on 88 melanoma samples and 102 colon cancer samples using validated antibodies for CMTM6^39^ (data not shown) or CD58 (Figure S5A). IHC analysis revealed widespread and largely overlapping expression of CMTM6 and CD58 in tumor cells in samples of both cancer types (Figures S5B, S5C, 4A, and 4C). Particularly, we observed that CD58 staining was largely restricted to areas with CMTM6 expression in 74 out of 88 melanomas. Moreover, we individually quantified the protein expression levels of CMTM6 and CD58 on tumor cells based on the IHC analysis. Both Spearman’s rank correlation test and chi-squared test showed a significant association between CMTM6 and CD58 expression in tumor cells across the analyzed tumor samples (Figures 4A–4D).

To assess the association between CMTM6 and CD58 expression and response to ICB therapies, we analyzed tumor biopsies obtained from 88 patients with advanced melanoma. These patients had not received prior treatment with anti-PD-1 and were subsequently treated with either anti-PD-1 or anti-PD1/anti-CTLA4 therapy. Patient characteristics are demonstrated (Table S1). We found no significant differences in CMTM6 or CD58 expression between anti-PD-1 and anti-PD-1/anti-CTLA4 treatment groups. However, our analysis revealed that higher levels of CMTM6 or CD58 expression were significantly associated with a favorable response to the ICB therapies (Figure 4E).

These findings highlight the relevance of CMTM6-mediated CD58 regulation in human tumors and suggest its potential critical role in modulating response to ICB therapies.

## DISCUSSION

Immune checkpoint pathways are often dysregulated in the TME, thereby promoting cancer progression and conferring resistance to immunotherapies. Decoding the molecular mechanisms that regulate immune checkpoint molecules is thus crucial for understanding immune regulation in cancer and, possibly, predicting response to immune checkpoint therapies and providing new therapeutic avenues.

The CD58−CD2 immune checkpoint constitutes a vital component in the immunological synapse that integrates signals for optimal T cell activation^26,44,45^ and cytolytic activities against tumor cells from various cancer types.^17,27,29–31^ Recent studies have linked the loss of CD58 to resistance in T cell-based cancer immunotherapies in both hematopoietic and solid cancers.^16,17^ However, aside from inactivating genetic mutations frequently found in lymphomas, the regulatory mechanisms underlying CD58 loss remain largely unclear.

Our findings, which demonstrate that CMTM6 positively regulates CD58 protein levels, combined with prior studies identifying CMTM6 as a positive regulator of PD-L1,^39,40^ offer molecular insights into how a single protein can control the expression of immune checkpoint ligands with opposing functions. Mechanistically, CMTM6 interacts with CD58 and PD-L1 on the cell membrane and protects them from lysosome-mediated protein degradation. Considering that CMTM6/PD-L1 interaction is crucial for maintaining PD-L1 stability, it is plausible that the CMTM6/CD58 interaction might fulfill a similar role in determining CD58 protein levels. This implies the potential value of individually targeting the interactions of CMTM6 and its partner proteins to modulate immune responses.

As PD-L1 and CD58 exert opposite effects on T cell activity, the net outcome of CMTM6 loss appears to be dependent on the balance between the two signals. In tumor cells with ectopic PD-L1 overexpression, CMTM6-mediated PD-L1 regulation predominantly modulates T cell activation in T cell-tumor cell cocultures.^39,40^ However, in tumor cells expressing only endogenous PD-L1, as tested in this study, CMTM6-mediated CD58 expression becomes the dominant factor. By comparing the functional outcome of individual or dual suppression of PD-L1 and CD58 in CMTM6-proficient and -deficient tumor cells in the T cell-tumor coculture, we revealed that CD58 expression is crucial for effective T cell-tumor cell interactions and response to PD-L1 inhibition. Moreover, we demonstrated the critical role of CMTM6-mediated CD58 expression in response to CAR-T treatment *in vivo*.

Notably, analysis of human tumor biopsies showed that both CMTM6 and CD58 are commonly expressed on tumor cells, and that their expression levels are positively correlated. Moreover, we observed that higher levels of CMTM6 and CD58 expression in tumor cells were associated with clinical benefit to ICB therapies. While this latter link provides only correlative evidence, we note that the frequent concurrent expression of CD58 and CMTM6 in human tumors provides support for the notion that the mechanistic effects here modeled *in vitro* and in a humanized mouse model may also be at play in human cancer.

In the accompanying study by Ho et al.,^46^ similar conclusions were drawn based on experiments conducted in different cell line and tumor models, with additional elaboration on the influence of CD58 expression on PD-L1 and vice versa, involving competing binding to CMTM6. On the other hand, our study provided further insights into the functional consequences of CMTM6-mediated regulation of PD-L1 and CD58. Additionally, we evaluated the clinical relevance of CMTM6 and CD58 protein expression in human cancers and explored their potential implications for ICB therapies.

Altogether, our study i) identified CMTM6 as a shared regulator and molecular partner of the CD58 and PD-L1 proteins, ii) revealed fundamental insight into CD58 regulation, iii) described CMTM6 as a modulator of immune responses through such regulations, and with a possible relevance of the CD58/CMTM6 interaction in human cancer lesions.

### Limitations of the study

In the presented *in vivo* experiment, CMTM6-proficient and -deficient tumor cells were mixed, followed by short-term treatment with CAR-T cells. While this experimental setting has been used to investigate T cell cytotoxicity toward target cells *in vivo*,^47–58^ it does not fully replicate actual CAR-T therapies. Although this experiment provided insights into the potential impact of the CMTM6-CD58 axis on tumor cell survival *in vivo*, further investigation is needed to understand how CMTM6 and CD58 expression on tumor cells modulates T cell activities. Besides, our results demonstrate a positive correlation between elevated levels of CMTM6 and CD58 expression in tumor cells and the clinical benefits in ICB treatment among a cohort of melanoma patients, suggesting potential clinical significance. Nevertheless, to validate them as predictive biomarkers, further prospective investigations involving larger patient cohorts are necessary.

## STAR★METHODS

### RESOURCE AVAILABILITY

#### Lead contact

Further information and requests for resources and reagents should be directed to and will be fulfilled by the lead contact, Chong Sun (c.sun@dkfz.de).

#### Materials availability

Materials generated in this study are available from the lead contact upon request.

#### Data and code availability

- The data generated during this study has been deposited at Mendeley Data (https://doi.org/10.17632/zcky9dypsr.1).
- For immunofluorescence signal analysis, a customized Matlab program is available at Mendeley Data. The DOI is listed in the key resources table.
- Any additional information required to reanalyze the data reported in this paper is available from the lead contact upon request.

### EXPERIMENTAL MODEL AND STUDY PARTICIPANT DETAILS

#### Cell lines

A375 and RKO cells were obtained from the American Type Culture Collection (ATCC), while 8505C, Ramos, OCI-AML2, and BJAB cells were obtained from the Deutsche Sammlung von Mikroorganismen und Zellkulturen GmbH (DSMZ). HAP1 cells were previously described by Carette et al. (2011). A375, RKO, 8505C, Ramos, and OCI-AML2 cells were cultured in RPMI 1640 medium (Thermo Fisher Scientific, Cat# 21875091), while HAP1 cells were cultured in IMDM (Thermo Fisher Scientific, Cat# 21980032). All media were supplemented with 10% fetal calf serum (FCS, Sigma-Aldrich, Cat# 10270-106), 100 U ml^−1^ penicillin-streptomycin (Thermo Fisher Scientific, Cat# 15140122), and GlutaMAX (Thermo Fisher Scientific, Cat# 15140122). IFNγ treatment was performed at a concentration of 50 ng ml^−1^ for 24 hours, unless otherwise indicated.

#### Mice

All animals were housed and treated in accordance with animal experimental protocols approved by the Ethical Committee of the Karlsruhe Regional Council. The experiments were performed in accordance with relevant local and national guidelines and regulations using 7–8-week-old male NOD.Cg-Prkdcscid Il2rgtm1Wjl/SzJ mice (The Jackson Laboratory). Mice were maintained under the Specific Pathogen-Free status (SPF) condition in the Central Animal Laboratory at the DKFZ Heidelberg and were randomly assigned to experimental groups.

#### Patient tumor samples

Human tissue collection for this study was carried out at the Netherlands Cancer Institute (melanoma) and Nanjing University (colon cancer). The collection of human tissues was conducted in accordance with the guidelines and regulations set forth by the Medical Ethical Review Board at respective institutions, and was approved for the study.

In this study, retrospective analysis was performed on existing materials obtained from patients who had already undergone treatment with the standard of care. In this context, a sample size calculation was not conducted. Patients with complete response (CR), partial response (PR), and stable disease (SD) lasting for 6 months or more were classified as having clinical benefit, while patients with progressive disease or with a SD for less than 6 months were categorized as having no clinical benefit.

### METHOD DETAILS

#### RP-nanoLC–MS/MS and data analysis

Parental and CMTM6-deficient 8505C cell pellets, which had been snap-frozen, were lysed through gentle homogenization using isotonic buffers supplemented with phosphatase inhibitor (PhosSTOP, Roche, Cat# 4906845001) and protease inhibitor (cOmplete mini EDTA-free, Roche, Cat# 11836170001). The efficiency of cellular disruption, exceeding 95%, was confirmed through microscopy. The cell lysate was then processed and analyzed using reverse-phase nano-flow liquid chromatography-tandem mass spectrometry (RP-nanoLC–MS/MS), following the methods described in the ref.^39^ Specifically, proteomics data were acquired using an UHPLC 1290 system (Agilent) coupled to an Orbitrap Q Exactive HF spectrometer (Thermo Scientific) for RP-nanoLC–MS/MS analysis. Peptides were initially trapped on a 2 cm × 100 μm Reprosil C18 pre-column (3 μm) and then separated on a 50 cm × 75 μm Poroshell EC-C18 analytical column (2.7 μm). Trapping was carried out for 10 min in 0.1 M acetic acid (solvent A), and elution was performed using 80% ACN in 0.1 M acetic acid (solvent B) with the following gradients: 10–40% solvent B in 155 min, 40–100% in 3 min, and finally 100% for 1 min. Flow was passively split to 300 nl min^−1^. MS data were obtained in data-dependent acquisition mode. Full scans were acquired in the m/z range of 375–1,600 at a resolution of 35,000 (m/z 400) with an AGC target of 3 × 10^6^. The top 15 most intense precursor ions were selected for HCD fragmentation performed at a normalized collision energy (NCE) of 25% after accumulation to a target value of 5 × 10^4^. MS/MS acquisition was performed at a resolution of 17,500.

For database search, raw files were processed using MaxQuant version 1.5.3.30 and searched against the human Swissprot database (version May 2016) using Andromeda. Cysteine carbamidomethylation was set as a fixed modification, while variable modifications of methionine oxidation and protein N-terminal acetylation, as well as up to 2 missed cleavages, were allowed. The false discovery rate (FDR) was restricted to 1% in both protein and peptide identification. Label-free quantification (LFQ) was performed with ’match between runs’ enabled. Data was analysed using Perseus software (v1.6.14). In each analysis, proteins quantified (LFQ) in two out of three replicates were log2 transformed and missing values were replaced individually for each sample from the normal distribution. Statistical differences were assessed by two-sided student’s t-test and double-filtered by fold change (up- or down-regulated >2-fold) and FDR-corrected p-values (<0.05) calculated using the permutation method with 250 iterations.

#### A FACS-based haploid genetic screen

HAP1 cells were mutagenized to generate a library of cells that carry insertional mutations. The resulting cells were expanded to approximately 1.5 × 10^9^ cells and subsequently dissociated using trypsin-EDTA (Life Technologies, Cat# 15090046), washed with PBS, and stained with anti-CD58-APC antibody (Thermo fisher scientific, Cat# 17-0578-42). Subsequently, cells were washed three times with PBS containing 1% FCS, passed through a 40 μm strainer (BD Falcon^™^, Cat# 352340), and subsequently fixed using BD fix buffer I (BD biosciences, Cat# 557870) for 10 min at 37°C, followed by a wash with PBS containing 1% FCS. The staining of the cells was finished with a final wash in PBS containing 10% FCS. The cell sorting and downstream processing were conducted as described in Brockmann et al.^60^

#### Generation of cell lines with knockdown, knockout, or overexpression of target genes

##### Knockout cell lines were generated using the CRISPR-Cas9 system.

To generate clonal knockout cells, parental cells were transfected with the pLentiCRISPRv2 vector (Addgene #52961) containing sgRNAs targeting the genes of interest. Following puromycin selection, single-cell clones were expanded and gene disruptions were confirmed through sequencing and western blot analysis. The sgRNA sequence CCGGGTCCTCCTCCGTAGTG was used to generate the 8505C CMTM6-knockout clone CMTM6 KO#1, the HAP1 CMTM6-knockout clone CMTM6 KO#1, the A375 CMTM6-knockout clone CMTM6 KO#6, and the RKO CMTM6-knockout clone CMTM6KO#1; the sgRNA sequence TCACAATG TACTTTATGTGG was used to generate the 8505C CMTM6-knockout clone CMTM6 KO#2, the HAP1 CMTM6-knockout clone CMTM6 KO#1, the A375 CMTM6-knockout clone CMTM6 KO#12, and the RKO CMTM6-knockout clone CMTM6 KO#2; the sgRNA sequence AAGCAATGTGCCTTTAAAAG was used to generate the HAP1 CD58-knockout clone.

To generate bulk knockout cells, transduction with lentiviral sgRNA (pLentiCRISPRv2, Addgene #52961; or pLentiGuide-Puro, Addgene # 52963 (or the derivatives pLentiGuide-Puro-GFP/pLentiGuide-Puro-BFP) + pLentiCas9-Blast, Addgene #52962) followed by antibiotics selection or cell sorting were carried out. For the derivative vectors-pLentiGuide-Puro-GFP/pLentiGuide-Puro-BFP, the coding sequences of GFP and BFP fluorescent proteins were individually cloned into the pLentiGuide-Puro vector with a P2A linker joining the puromycin resistance element.

The sgRNA sequence TCACAATGTACTTTATGTGG was used to generate bulk CMTM6-knockout Ramos, BJAB, OCI-AML2, A375, and 8505C cells; the sgRNA sequence CACCACCAATTCCAAGAGAG was used to generate bulk PD-L1-knockout cells; the sgRNA sequence GCAGCAGGCAGACCACGCTG was used to generate bulk CD58-knockout cells. The OCI-AML2 cells used in the *in vivo* experiment were generated using a non-targeting control sgRNA GTATTACTGATATTGGTGGG (gNT-GFP cells) and a CMTM6-targeting sgRNA TCACAATGTACTTTATGTGG (gCMTM6-BFP cells).

Lentiviral shRNA vectors were generated using the hairpin sequences retrieved from The RNAi Consortium shRNA Library (https://portals.broadinstitute.org/gpp/public/gene/search) and listed in the key resources table.

To achieve ectopic expression of CD58 (ENSEMBL: ENST00000369489.10) or CMTM6 (ENSEMBL: ENST00000205636.4), the coding sequences were obtained from Ensembl, codon-optimized, synthesized by Twist Biosciences, and cloned into a pCDH-CMV-MCS-EF1-Hygro vector or its derived vector, pCDH-CMV-MCS-EF1-Puro or pCDH-CMV-MCS-mPGK-Blast.

To produce lentivirus, the transfer plasmids containing the gRNA, shRNA, or ORF sequences were transfected into HEK293T cells along with packaging plasmids (Addgene plasmid #12260 psPAX2, #12259 pMG2.G) by PEI-MAX. The medium was refreshed after 24 hours. 48 hours after the transfection, the supernatant containing lentivirus was collected and used for transduction.

#### Flow cytometry

Cell suspensions were stained with antibodies as indicated. Dead cells were excluded based on 4,6-diamidino-2-phenylindole (DAPI) incorporation, or using the LIVE / DEAD ^™^ Fixable Near-IR Dead Cell Stain Kit (Thermo Fisher Scientific, Cat# L10119). Washing and reagent dilutions were performed in PBS containing 2% FCS and 0.09% sodium azide (NaN_3_) except that the dilution of LIVE / DEAD^™^ Fixable Near-IR staining reagent was in PBS. Data acquisition was performed on a BD LSR Fortessa cytometer (BD Biosciences) interfaced to the FACS-Diva software system, and analyzed by FlowJo software.

#### Western blot

Cells for western blot analysis were seeded in 6-well plates and treated as described in the figure legends. Proteinase inhibitor (cOmplete mini EDTA-free, Roche, Cat# 11836170001) was freshly dissolved and added to RIPA lysis buffer. The cells were washed with PBS before being lysed by RIPA lysis buffer. The lysate was collected by the cell lifter (Corning, Cat# CLS3008-100EA) and kept on ice for 30 min, then centrifuged at 14000rpm for 15min at 4°C. The supernatant was quantified by BCA protein assay kit (Thermo Scientific, Cat# 71285-3). The normalized samples were loaded to NuPAGE Gel Electrophoresis System (Invitrogen, Cat# WG1403BOX) and processed according to the manufacturer’s instructions.

#### (Cell surface) immunoprecipitation (IP)

Cells for (surface) immunoprecipitation experiments were seeded and cultured in 15-cm dishes. For the cell harvesting, cells were washed with cold PBS buffer and then detached from culture plates by TrypLE Express (Thermo Fisher Scientific, Cat# 12604013), followed by washing with PBS buffer containing 1% FBS. 5 million cells were used per immunoprecipitation reaction for the regular IP in whole-cell lysate and 2 million cells per reaction for cell surface IP.

For the regular IP, cell pellets were lysed in digitonin buffer (1% digitonin (Millipore, Cat# 300410), 50 mM Tris-HCl pH 7.5, 150 mM NaCl) for 30 minutes on ice. After centrifugation, the supernatant was incubated with antibodies as indicated (2μg antibodies per reaction) for 2h at 4°C. The antibody/lysate solution was subsequently incubated with Dynabeads^™^ Protein G (25μl per reaction) for Immunoprecipitation (Thermo Fisher Scientific, Cat# 10003D) for 2h at 4°C.

For the cell surface IP, cell pellets were first resuspended in PBS buffer containing 1%FCS and antibodies targeting the proteins as indicated (2 μg antibodies per reaction). After incubation on a rotator for 2 hours at 4°C, cells were washed to remove unbound antibodies, followed by cell lysis in digitonin buffer (1% digitonin (Millipore, Cat# 300410), 50 mM Tris-HCl pH 7.5, 150 mM NaCl) for 30 minutes on ice. After centrifugation, the supernatant was subsequently incubated with Dynabeads^™^ Protein G (25μl per reaction) for Immunoprecipitation (Thermo Fisher Scientific, Cat# 10003D) for 2h at 4°C.

After the incubation, the beads were washed 2 times using digitonin buffer, and the elution was performed using Bolt^™^ LDS Sample Buffer (Thermo Fisher Scientific, Cat# B0007) supplemented with Sample Reducing Agent (Thermo Fisher Scientific, Cat# B0009). The final elute was subjected to western blot analysis.

#### Reverse transcription-quantitative polymerase chain reaction (RT-QPCR)

Total RNA was extracted using the RNeasy Plus Mini Kit (Qiagen, Cat# 74134), following the manufacturer’s instructions. Subsequently, cDNA synthesis was performed using the Maxima First Strand cDNA Synthesis Kit (Thermo Fisher Scientific, Cat# K1641). Amplification of mRNA transcripts was carried out using gene-specific primers and SYBR Green master mix (Thermo Fisher Scientific, Cat# A25742) and measured using a ViiA 7 Real-Time PCR System (ABI). To quantify the mRNA levels, the relative expression of each gene was normalized to GAPDH.

#### *In vitro* differentiation of progenitor cells into DC

The Lin-CD34+c-Kit+ cells were sorted from peripheral blood mononuclear cells (PBMCs) obtained from healthy donors by flow cytometry. The sorted cells were seeded at a density of 1000 to 2000 cells per well in round-bottom 96-well plates (BD Falcon, Cat# 351172) and cultured at 5% CO2 and 37°C in Iscove modified Dulbecco medium (IMDM, Gibco) supplemented with 10% fetal calf serum (FCS, Sigma) and relevant cytokines. To induce differentiation into dendritic cells (DCs), the cells were cultured for 2 to 3 weeks in medium containing 100 ng/mL of recombinant human (rh) Flt3 ligand (Flt3L, Miltenyi Biotec Cat#170-076-132), 0.5 ng/mL of rh macrophage colony-stimulating factor (M-CSF, Miltenyi Biotec Cat#170-076-170), 1 ng/mL of granulocyte-macrophage colony-stimulating factor (GM-CSF, Miltenyi Biotec Cat#170-076-112), 5 ng/mL of rh thrombopoietin (TPO, Miltenyi Biotec Cat#170-076-134), 10 ng/mL of rh interleukin-3 (IL-3, Miltenyi Biotec Cat#170-076-110), and 5 ng/mL of rh stem cell factor (SCF, Miltenyi Biotec Cat#170-076-149). All growth factors were purchased from Miltenyi Biotec. Mesenchymal stromal cells were irradiated with 8Gy before being used as feeder layers at a density of 3000 cells/well.

#### Immunofluoresence imaging

3×10^4^ A375 or 8505C cells were seeded in 24-well plates with one coverslip per well. After two days, the coverslips were rinsed with PBS buffer and fixed using 50 μL of fixation buffer (Bioscience intracellular fixation & permeabilization buffer set, Thermo Fisher Scientific Cat# 88-8824-00) directly on the coverslips. The cells were then incubated at room temperature for 20 minutes. Next, 30 μL of primary antibodies against CMTM6 and CD58, prepared in permeabilization buffer (Thermo Fisher Scientific, Cat# 88-8824-00), were added to each coverslip and incubated at room temperature for 3 hours. Following that, the coverslips were washed three times with PBS buffer and incubated with fluorophore-conjugated secondary antibodies, prepared in 3% BSA, at room temperature for 2 hours. After another three washes with PBS buffer, the coverslips were incubated with one of the fluorophore-conjugated antibodies (in 3% BSA) targeting EEA1, LAMP1, or TFRC at room temperature for 1 hour. Finally, the coverslips were mounted with Prolong Glass Antifade Mountant (Thermo Fisher Scientific, Cat# P36984), and images were captured using a Zeiss LSM 780 SD confocal microscope.

For immunofluorescence signal analysis, a customized Matlab program (available at https://github.com/YGanLab/Image_ColorChannel_Intensity Li, X., & Gan, Y. (2023) and Mendelay Data the link is available in the key resources table). Calculate intensity of medical images over color-channels’) was used. The program separated the signals using individual color channels representing EEA1, TFRC, LAMP1, CD58, CMTM6, and DAPI. The signal intensity across image pixels for a specific marker indicating the region of interest was automatically calculated for each channel.

The colocalization ratio, C, was determined by comparing the measurements of the marker in two channels, $M_{1}$ and $M_{2}$, using the equation $C=\frac{I\left(M_{1}\right)}{I\left(M_{2}\right)}*100\%$, where $I\left(M_{1}\right)$ and $I\left(M_{2}\right)$ were the summation of intensity over pixels for channel $M_{1}$ and channel $M_{2}$, respectively.

#### Protein stability assay

A375 cells were detached from culture plates using TrypLE and then washed twice with PBS. The cells were labeled with either anti-CD58-APC (TS2/9, Biolegend, Cat# 330918), anti-CD274-APC (29E.2A3, Biolegend, Cat# 393610), or anti-HLA-A,B,C-APC (W6/32, Biolegend, Cat# 311410). After two additional washes, the cells were resuspended in complete medium and incubated at 37°C in the presence or absence of lysosome inhibitor and proteasome inhibitor, as specified. The cells were collected at indicated time points, and the APC signals were measured by flow cytometry.

#### Generation of MART-1 TCR-T cells and CD33 CAR-T cells

MART-1-specific (TCR clone #1D3) CD8^+^ T cells were generated following the protocol outlined in Jorritsma et al. Blood (2007). For the CD33 CAR-T cell generation, 7 million HEK293T cells were seeded in a 10 cm cell culture dish one day prior to transfection. The transfection involved a plasmid mixture comprising 7.5 μg of the retroviral transfer plasmid pMP71_CD33Hul95-CD28zCAR (the CAR construct is described as "CD33Hul95-CD28z" in the patent WO2019178382A1), 4.5 μg of pGag/pol, 2.94 μg of pVSV-G, and 1.92 μg of pAdvanced plasmids. This mixture was transfected using 50 μL of PEI-MAX reagent. After the initial 24 hours, the culture medium was refreshed, and then the virus supernatant was harvested after another 24 hours. Subsequently, 1 mL of the supernatant was added to a 24-well plate that had been treated with retronectin at a concentration of 5 μg/mL overnight at 4°C. The plate was then centrifuged at 3500 rpm for 2 hours with a deacceleration rate of zero.

Next, 2.5×10^5^ T cells, which had been pre-activated for 48 hours using Dynabeads^™^ Human T-Activator CD3/CD28, were resuspended in 500 μL of T cell medium. The T cell medium consisted of RPMI1640 supplemented with 10% FBS, 5 ng/mL IL-15, and 50U/mL IL-1. The resuspended T cells were then added to the retronectin and retrovirus -coated 24-well plate. The next day, an additional 500 μL of fresh T cell medium was added to the wells. The efficiency of T cell transduction was assessed by flow cytometry before the cells were used in the coculture experiment.

#### Cocultures of MART-1-specific CD8^+^ T cells and MART-1-loaded tumor cells

For each well of a 6-well plate, 1–2×10^5^ A375 or 8505C cells were seeded, and the MART-1 ELA mutant epitope peptide (ELAGIGILTV) dissolved in complete RPMI medium was incubated with tumor cells at a final concentration of 10 ng/mL. After 2-hour incubation, the cells were washed with fresh medium to remove excess peptide. Alternatively, a gene fragment encoding the MART-1 epitope followed by P2A-RFP was cloned into a lentiviral vector, and the resulting virus was used to transduce tumor cells to enable the MART-1 antigen presentation. MART1-specific CD8^+^ T cells and MART1-loaded tumor cells were then mixed at a ratio of 1:4. After incubation at 37°C and 5% CO2 for the specified durations, T cells were collected and stained for measuring T cell activation markers using flow cytometry. After the T cell removal, tumor cell viability was determined using the CellTiter Blue Assay (Promega, Cat# G8020).

#### Mouse xenograft experiment

CMTM6-proficient and -deficient OCI-AML2 cells were generated using a non-targeting (gNT-GFP) and a CMTM6-targeting sgRNA constructs (gCMTM6-BFP), respectively. These cells labeled with different fluorescent proteins were prepared for the transplantation into 7–8-week-old male NSG mice. To facilitate human CAR-T cell survival in mice, 6.5x10^5^ IU of recombinant human IL-2 was administered intraperitoneally (i.p.) 12 hours prior to OCI-AML2 cell injection in the CAR-T cell treatment group. In the anti-CD58 antibody treatment group, 100μg of anti-human CD58 blocking antibody was also administered (i.p.). For the anti-CD58 antibody treatment group, the tumor cells were pre-treated with the antibody at a concentration of 5 ng/μL for 30 minutes at 37°C to achieve more comprehensive CD58 blockade before the transplantation. For the untreated control group, tumor cells were incubated in PBS.

The CMTM6-proficient (gNT-GFP) and CMTM6-deficient (gCMTM6-BFP) OCI-AML2 cells were mixed at a 1:1 ratio. CD33-CAR-T cells were prepared as described in the section “generation of MART-1 TCR-T cells and CD33 CAR-T cells” and added to the mixed OCI-AML2 cells on ice right before the intravenous injection for the T cell treatment group, at the specified T cell-tumor cell ratios. A total of 3 million OCI-AML2 cells (with or without CD33 CAR-T cells) were injected intravenously into the tail vein of NSG mice in a volume of 150 μL per mouse. After seven hours from the OCI-AML2 injection, the mice were sacrificed by CO_2_ inhalation, and blood was collected via heart puncture. The ratio of GFP and BFP-positive AML cells was analyzed using flow cytometry.

#### Immunohistochemistry

Immunohistochemistry of the FFPE tumor samples from human melanoma was performed on a BenchMark Ultra autostainer (Ventana Medical Systems). Briefly, paraffin sections were cut at 3 μm, heated at 75°C for 28 minutes and deparaffinized in the instrument with EZ prep solution (Ventana Medical Systems). Heat-induced antigen retrieval was carried out using Cell Conditioning 1 (CC1, Ventana Medical Systems) for 64 minutes at 95°C. CD58 was detected using clone 126 (1/800 dilution, 1 hour at RT, Novus Biologicals, Cat. No. NBP2-90097) and CMTM6 hybridoma supernatant, clone 6B2 at a dilution of 1/3200 (stock concentration: 1mg/ml), also incubated for 1 hour at room temperature. Bound antibody was detected using the OptiView DAB Detection Kit (Ventana Medical Systems). Slides were counterstained with Hematoxylin and Bluing Reagent (Ventana Medical Systems). A PANNORAMIC^®^ 1000 scanner from 3DHISTECH was used to scan the slides at a 40x magnification.

The formalin-fixed paraffin-embedded human colon cancer sample was baked at 62°C for 1 hour and soaked with fresh xylene (Sinopharm Chemical Reagent Co., Ltd, Cat# 10023418) for 3 times, each time for 20 minutes to remove paraffin. Next, the slice was hydrated by immersion in ethanol (Sinopharm Chemical Reagent Co., Ltd, Cat# 100092683) at the following concentrations: 100%, 100%, 95%, 95%, 70% for 1 minute each. After washing with tap water for 5 minutes, the slice was rinsed with distilled water. 100 μL of hydrogen peroxide blocking solution was added to each tablet and incubated for 10 minutes at room temperature. After washing with PBS for 3 times, the slice was boiled in the antigen retrieval solution for 15 minutes, followed by another 15 minutes of holding at the temperature, and then cooled down naturally. The slide was washed with PBS for 3 times, 100 μL of 5% BSA (Sigma, Cat# B2064) was added to block the slice for 20 minutes, and then the BSA was removed. 50 μL of primary CD58 (clone 126, dilution 1:300) (SinoBiological, Cat# 12409-R126) or CMTM6 (clone RCT6, dilution 1:800) (Absea, Cat# K06153M01D06C) antibody was added to the slice and incubated overnight. The slice was kept at room temperature for 1 hour and then washed with PBS for 3 times. 50 μL of secondary antibody (DAKO, Cat# K5007) was added and kept at 37°C for 30 minutes. After washing with PBS for 3 times, DAB (DAKO, Cat# K5007) was added to the slice to develop the staining and monitored under the microscope. The DAB was immediately rinsed off with tap water when yellow particles or flaky precipitates appeared, re-stained with hematoxylin (Runnerbio, Cat# Bry-0001-01) for 30 seconds, and rinsed with tap water for 5 minutes. The slice was differentiated with hydrochloric acid alcohol (Runnerbio, Cat# Bry-0001-03) for 1 second, followed by washing with tap water for 10 minutes. The slice was dehydrated with the following ethanol concentration series: 70%, 95%, 95%, 100%, 100%, each for 3 minutes. Next, the slice was soaked in xylene for 5 minutes for transparency and then sealed with neutral resin sealing gum (Sinopharm Chemical Reagent Co., Ltd, Cat# 10004160).

To calculate the IHC score for each sample, a numerical value was first assigned to each sample based on the overall staining intensity of all cancer cells with a positive signal. No expression was assigned a value of 0, while weak, moderate, strong, and very strong expressions were assigned as 1, 2, 3, and 4, respectively. This intensity value was multiplied by the proportion of positive cancer cells relative to the total number of cancer cells in that sample to yield an IHC score representing the overall expression level of CMTM6 and CD58 in each sample. This scoring method was applied to assess the expression levels of both CMTM6 and CD58 in both colon cancer and melanoma samples.

In parallel, H-score scoring method was also used by an independent pathologist (negative expression was denoted as 0, weak expression as 1+, moderate expression as 2+, and strong expression as 3+) to assess the melanoma samples. In this case, the final IHC scores by two independent pathologists were normalized to a scale of 0–300, and the average score was reported.

#### Melanoma patients received anti-PD-1 or anti-PD-1/anti-CTLA-4 treatment

In this retrospective analysis, melanoma patients were treated according to standard medical care. A total of 88 pre-treatment biopsies were selected from anti-PD1-naïve patients who thereafter were treated with either anti-PD1 monotherapy or a combination therapy of anti-PD1 and anti-CTLA-4. The patients were then divided into two groups based on the observed response to the treatment: those who achieved clinical benefit and those who did not. The group of patients with clinical benefit included individuals who achieved a complete response, partial response, or stable disease lasting for at least 6 months. On the other hand, the group of patients without clinical benefit consisted of those who had progressive disease or stable disease for less than 6 months.

To assess the protein expression levels of CMTM6 and CD58, comparisons were made between patients with clinical benefit and those without clinical benefit using the Wilcoxon-rank sum test. The statistical analysis was performed using the R programming language (version 4.2.2).

### QUANTIFICATION AND STATISTICAL ANALYSIS

Statistical analysis was performed, and the outcomes were presented as described in figure legends and STAR Methods. Specifically, unpaired student’s t-test in Figures 1A, 1B, 2A–2C, and S1G–S1J, two-way ANOVA test (with Tukey’s multiple comparisons test for multiplicity adjusted P values) in Figures 1H–I, 3B, 3D, 3E–3G, S3A, S3B, S3E–S3I, and S4B–S4C, one-way ANOVA (with Tukey’s multiple comparisons test for multiplicity adjusted P values) in Figures S1K, S1L, S2B, and S2D, and S4E, two-sided Fisher’s exact test (with Benjamini-Hochberg false discovery rate correction) in Figure 1E, spearman correlation analysis in Figures 4A and 4C, chi-square test in Figures 4B and 4D, and Wilcoxon-rank sum test in Figure 4E were performed. All ANOVA test, student’s t-test, Spearman correlation, and Chi-square analyses were performed using Prism (version 9.5.1) software, while the others were analyzed using R studio. All ANOVA and student’s t-test analysis were based on triplicate data and presented as mean ± standard, unless otherwise specified in the figure legends. In Figure 4E, it was observed that the IHC scores of CD58 did not follow a normal distribution, as indicated by the Shapiro-Wilk test. Consequently, the Wilcoxon-rank sum test was used for the analyses related to the association between the levels of CMTM6 or CD58 and clinical benefit. No further assessment of the distribution of data were conducted.

## Supplementary Material

S1

S2

S3

S4

S5

Table S1

## Figures and Tables

**Table:** KEY RESOURCES TABLE

REAGENT or RESOURCE	SOURCE	IDENTIFIER
Antibodies		

Mouse anti-human CD58 (TS2/9)	Biolegend	Cat# 330924; RRID: AB_2832649
Rabbit anti-human CD58 (EP15041)	Abcam	Cat# ab196648; RRID: AB_2943462
Goat anti-human CD58	R&D systems	Cat# AF1689; RRID: AB_354933
Rabbit anti-human CD58 (for IHC)	Novus Biologicals	Cat# NBP2-90097; RRID: AB_2943463
Rabbit anti-human CD58 (for IHC)	SinoBiological	Cat# 12409-R126; RRID: AB_2860423
Mouse anti-human PD-L1 (405.9A11)	Cell signaling technology	Cat# 29122; RRID: AB_2798970
Rabbit anti-human PD-L1 (E1L3N)	Cell signaling technology	Cat# 13684S; RRID: AB_2687655
Mouse anti-human CMTM6 (RCT6)	Absea	Cat# K06143M03F06C; RRID: AB_2943464
Mouse anti-human CMTM6 (6B2)	This study (Generated by Absea)	RRID: AB_2943461
Rabbit anti-human CMTM6	Atlas Antibodies	Cat# HPA026980; RRID: AB_10602801
Mouse anti-human HSP90 (F-8)	Santa Cruz	Cat# sc13119; RRID: AB_675659
Goat anti-Mouse IgG (H + +L)	Jackson ImmunoResearch	Cat# 115-035-146; RRID: AB_2307392
Goat anti-Rabbit IgG (H+L)	Jackson ImmunoResearch	Cat# 111-035-144; RRID: AB_2307391
Goat anti-Mouse light chain specific	Jackson ImmunoResearch	Cat# 115-005-174 RRID: AB_2338460
Mouse anti-Rabbit light chain specific	Jackson ImmunoResearch	Cat# 211-032-171; RRID: AB_2339149
Mouse anti-Goat IgG	Santa Cruz	Cat# sc2354; RRID: AB_628490
Donkey anti-Goat IgG (H+L)	Invitrogen	Cat# A16005; RRID: AB_2534679
humanized anti-human PD-L1	Genentech	RRID: AB_2943467
Mouse anti-human CD137-PE/Cyanine7 (4B4-1)	Biolegend	Cat# 309818, RRID: AB_2207741
Mouse anti-human CD69-APC/FireTM 750 (FN50)	Biolegend	Cat# 310946; RRID: AB_2616709
Mouse anti-human CD3-Alexa Fluor 700 (SK7)	Biolegend	Cat#344822; RRID: AB_2563420
Mouse anti-human CD2-APC (RPA-2.10)	Biolegend	Cat# 300214; RRID: AB_10895925
Mouse anti-human CD28-FITC (CD28.2)	Biolegend	Cat# 302906; RRID: AB_314308
Mouse anti-human CD279-PE (EH12.2H7)	Biolegend	Cat# 329906; RRID: AB_940483
Mouse anti-human CD274-PE (29E.2A3)	Biolegend	Cat# 329706; RRID: AB_940368
Mouse anti-human CD58-APC (TS2/9)	Biolegend	Cat# 330918; RRID: AB_2650886
Mouse anti-human CD58 BUV395 (Clone L306.4)	BD Biosciences	Cat# 745571; RRID: AB_2743091
Mouse anti-human CD274-APC (29E.2A3)	Biolegend	Cat# 329708; RRID: AB_940360
Mouse anti-HLA-A, B, C-APC (W6/32)	Biolegend	Cat# 311410; RRID: AB_314879
Rat anti-human IL-2-PE/Cy7 (MQ1-17H12)	Biolegend	Cat# 500326; RRID: AB_2125593
Mouse anti-human TNF-α-Brilliant Violet 785 (MAb11)	Biolegend	Cat# 502948; RRID: AB_2565858
Mouse anti-human IFN-γ-Brilliant Violet 421 (4S.B3)	Biolegend	Cat# 502532; RRID: AB_2561398
Mouse BD HorizonTM Customs BYG584-P CMTM6 (6B2)	This study (Fluorophore conjugation by BD)	RRID: AB_2943461
Mouse IgG1, Isotype Control antibody (MOPC-21)	Biolegend	Cat# 400102; RRID: AB_2891079
Humanized Human IgG1, Isotype Control antibody (QA16A12)	Biolegend	Cat# 403502; RRID: AB_2927629
Goat anti-Rabbit IgG (H+L) Highly Cross-adsorbed Secondary antibody, Alexa Fluor^™^ Plus 488	Invitrogen	Cat# A32731; RRID: AB_2633280
Goat anti-Mouse IgG (H+L) Cross-adsorbed Secondary antibody, Alexa Fluor 555	Invitrogen	Cat# A21422; RRID: AB_2535844
Goat anti-Rabbit IgG (H+L) Highly Cross-adsorbed Secondary antibody, Alexa Fluor Plus 647	Invitrogen	Cat# A32733; RRID: AB_2633282
Human/Mouse/Rat EEA1 Alexa Fluor^®^ 488-conjugated antibody	R&D systems	Cat# IC8047G; RRID: AB_2943466
Alexa Fluor 647 Mouse anti-Human Transferrin R (CD71) (OKT9)	BD Biosciences	Cat# 566724; RRID: AB_2869830
Alexa Fluor 647 anti-human CD107a (LAMP-1) antibody (H4A3)	Biolegend	Cat# 328611; RRID: AB_1227507

Bacterial and virus strains		

One Shot^™^ Stbl3^™^ Chemically Competent E. coli	ThermoFisher Scientific	Cat # C737303

Biological samples		

Human melanoma tumor material	Netherlands Cancer Institute	N03LAM/CFMBP547
Human colon tumor material	Nanjing Drum Tower hospital/Nanjing University	ZDX22001

Chemicals, peptides, and recombinant proteins		

Recombinant Human CD2 Fc Chimera Protein	R&D systems	1856-CD-050
MART-1 ELA mutant epitope peptide	This study	N/A
Digitonin	Millipore	Cat# 300410
Prolong Glass Antifade Mountant	Thermo Fisher Scientific	Cat# P36984
Proteinase inhibitor	Roche	Cat# 11836170001
Proleukin S (IL-2)	Novartis	N/A

Critical commercial assays		

First Strand cDNA Synthesis Kit	Thermo Fisher Scientific	Cat# K1641
Bioscience intracellular fixation & permeabilization buffer set	Thermo Fisher Scientific	Cat# 88-8824-00
LIVE / DEAD ^™^ Fixable Near-IR Dead Cell Stain Kit	Thermo Fisher Scientific	Cat# L10119

Experimental models: Cell lines		

RKO	ATCC	N/A
A375	ATCC	N/A
8505C	DSMZ	N/A
Ramos	DSMZ	N/A
BJAB	DSMZ	N/A
OCI-AML2	DSMZ	N/A
HAP1	Carette et al.^59^	N/A

Experimental models: Organisms/strains		

NOD.Cg-Prkdcscid Il2rgtm1Wjl/SzJ	The Jackson Laboratory	RRID:IMSR_JAX:005557

Oligonucleotides		

Human-GAPDH-qPCR-F: AAGGTGAAGGTCGGAGTCAA	Sigma	N/A
Human-GAPDH-qPCR-R: AATGAAGGGGTCATTGATGG	Sigma	N/A
Human-CD58-qPCR-F: GACACTGTGTCAGGTAGCCTCA	Sigma	N/A
Human-CD58-qPCR-R: GCACAAGTTAGTGTGGGAGATGG	Sigma	N/A

Recombinant DNA		

Plasmid: pMD2.G	Addgene	Cat #12259
Plasmid: psPAX2	Addgene	Cat #12260
Plasmid: pCMV-VSV-G	Addgene	Cat #8454
Plasmid: gag/pol	Addgene	Cat #14887
Plasmid: padVantage	Promega	E171A
Plasmid: pLentiCRISPRv2	Addgene	Cat #52961
Plasmid: pLentiCas9-Blast	Addgene	Cat #52962
Plasmid: plentiGuide-Puro	Addgene	Cat #52963
Plasmid: pLentiGuide-GFP-2A-GFP	This study	N/A
Plasmid: pLentiGuide-BFP-2A-BFP	This study	N/A
Plasmid: pLentiCRISPRv2-sgNT (non-targeting)(GTATTACTG ATATTGGTGGG)	This study	sgRNA sequence is extracted from Human CRISPR Knockout Pooled Library (Brunello) Addgene: 73178
Plasmid: pLentiCRISPRv2-sgCMTM6#1 (CCGGGTCCTCCTCCGTAGTG)	This study	sgRNA sequence is extracted from Human CRISPR Knockout Pooled Library (Brunello) Addgene: 73178
Plasmid: pLentiCRISPRv2-sgCMTM6#2 (TCACAATGTACTTTATGTGG)	This study	sgRNA sequence is extracted from Human CRISPR Knockout Pooled Library (Brunello) Addgene: 73178
Plasmid: pLentiCRISPRv2-sgCD58(GCAGCAGGCAGACCACGCTG)	This study	sgRNA sequence is extracted from Human CRISPR Knockout Pooled Library (Brunello) Addgene: 73178
Plasmid: pLentiCRISPRv2-sgPD-L1(CACCACCAATTCCAAGAGAG)	This study	sgRNA sequence is extracted from Human CRISPR Knockout Pooled Library (Brunello) Addgene: 73178
Plasmid: pLentiGuide-GFP-2A-Puro-sgNT (non-targeting) (GTATTACTGATATTGGTGGG)	This study	sgRNA sequence is extracted from Human CRISPR Knockout Pooled Library (Brunello) Addgene: 73178
Plasmid: pLentiGuide-BFP-2A-Puro-sgCMTM6#2 (TCACAATGT ACTTTATGTGG)	This study	sgRNA sequence is extracted from Human CRISPR Knockout Pooled Library (Brunello) Addgene: 73178
Plasmid: pLKO.1 puro	Addgene	Cat #8453
shCMTM6 hairpin sequence (CCGGCCTTTCTTCTGAGTCTCCT TACTCGAGTAAGGAGACTCAGAA GAAAGGTTTTTTG)	The RNAi Consortium shRNA Library	TRCN0000127888
shCD58-1 hairpin sequence (CCGGGCCTCACTATCTACAACTT AACTCGAGTTAAGTTGTAGATAGT GAGGCTTTTTG)	The RNAi Consortium shRNA Library	TRCN0000057543
shCD58-2 hairpin sequence (CCGGGCGGTCATTCAAGACACG GGTCTCGAGATCTGTGTCTTGAAT GACCGCTTTTTG)	The RNAi Consortium shRNA Library	TRCN0000057544
shCD58-3 hairpin sequence (CCGGGTGCTGTATATGAATGGT ATTCTCGAGAATACCATTCATAT ACAGCACTTTTTG)	The RNAi Consortium shRNA Library	TRCN0000057545
shCD58-4 hairpin sequence (CCGGGTGTTGTGTATGGGAATG TAACTCGAGTTACATTCCCATAC ACAACACTTTTTG)	The RNAi Consortium shRNA Library	TRCN0000057546
shCD58-5 hairpin sequence (CCGGGCATTGACTAATGGAAGC ATTCTCGAGAATGCTTCCATTAGT CAATGCTTTTTG)	The RNAi Consortium shRNA Library	TRCN0000057547
shCD58-6 hairpin sequence (CCGGACGTAACTCAACCAGTAT ATACTCGAGTATATACTGGTTGA GTTACGTTTTTTG)	The RNAi Consortium shRNA Library	TRCN0000312623
shCD58-7 hairpin sequence (CCGGTACTCTTAGCAATCCATTA TTCTCGAGAATAATGGATTGCTA AGAGTATTTTTG)	The RNAi Consortium shRNA Library	TRCN0000312555
shCD58-8 hairpin sequence (CCGGGAAGACAACAGCATAACT AAACTCGAGTTTAGTTATGCTGT TGTCTTCTTTTTG)	The RNAi Consortium shRNA Library	TRCN0000312624
Plasmid: pCDH-CMV-MCS-EF1-Hygro	System Bioscience	CD515B-1
Plasmid: pCDH-CMV-MCS-EF1-Puro	System Bioscience	CD510B-1
Plasmid: pCDH-CMV-MCS-mPGK-Blast	This study	N/A
Plasmid: pMP71-CD33Hul95-CD28zCAR	This study	N/A
Plasmid: pCDH-Hygro-CD58 (ENST00000457047.6)	This study	N/A
Plasmid: pCDH-Puro-CMTM6 (ENST00000205636.4)	This study	N/A
Plasmid: pCDH-MART-1epi-2A-RFP	This study	N/A

Software and algorithms		

Matlab	The MathWorks	RRID: SCR_001622
FlowJo v10	FlowJo, LLC	RRID: SCR_008520
R (v 4.2.2)	R project	RRID: SCR_001905
ggplot2	Wickham	https://ggplot2.tidyverse.org
Graphpad prism	Dotmatics	RRID:SCR_002798
Zeiss ZEN 3.7	Zeiss	RRID:SCR_013672
Image Lab V6.1	Bio-rad	http://www.bio-rad.com/en-us/sku/1709690-image-lab-software; RRID:SCR_014210
Riorender	Biorender	https://www.biorender.com/
Image_ColorChannel_Intensity	This paper	Mendeley Data: https://doi.org/10.17632/zcky9dypsr.1
**Deposited data**		
Data generated in this study	This paper	Mendeley Data: https://doi.org/10.17632/zcky9dypsr.1

## Highlights

- CMTM6 maintains the expression of CD58
- CMTM6 co-regulates and interacts with both CD58 and PD-L1
- Tumor cell-intrinsic CMTM6 and CD58 are important for antitumor T cell response
