# Supplementary material for: CMTM6 shapes antitumor T cell response through modulating protein expression of CD58 and PD-L1
Source: Cancer Cell. Author manuscript; Available in PMC 2024 May 23. (PMC11113010; doi:10.1016/j.ccell.2023.08.008)
Supplement: S1 [file NIHMS1981828-supplement-S1.pdf]

Cancer Cell, Volume *41*

Supplemental information

CMTM6 shapes antitumor T cell response through modulating protein expression of CD58 and PD-L1

Beiping Miao, Zhaoqing Hu, Riccardo Mezzadra, Lotte Hoeijmakers, Astrid Fauster, Shangce Du, Zhi Yang, Melanie Sator-Schmitt, Helena Engel, Xueshen Li, Caroline Broderick, Guangzhi Jin, Raquel Gomez-Eerland, Lisette Rozeman, Xin Lei, Hitoshi Matsuo, Chen Yang, Ingrid Hofland, Dennis Peters, Annegien Broeks, Elke Laport, Annika Fitz, Xiyue Zhao, Mohamed A.A. Mahmoud, Xiujian Ma, Sandrine Sander, Hai-kun Liu, Guoliang Cui, Yu Gan, Wei Wu, Yanling Xiao, Albert J.R. Heck, Wenxian Guan, Scott W. Lowe, Hugo M. Horlings, Cun Wang, Thijn R. Brummelkamp, Christian U. Blank, Ton N.M. Schumacher, and Chong Sun

Figure S1

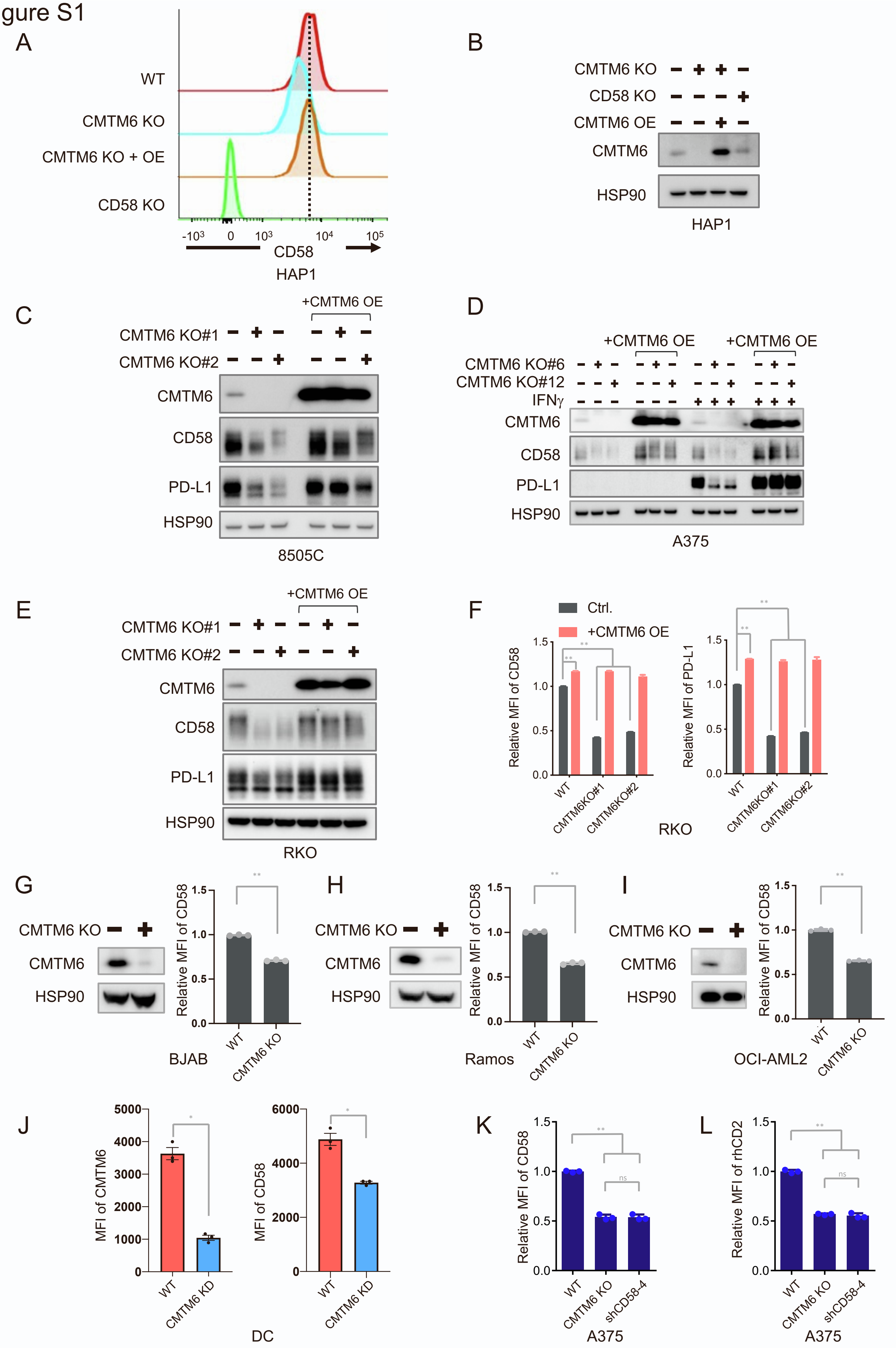
A
WT
CMTM6 KO
CMTM6 KO + OE
CD58 KO
CD58
HAP1
B
CMTM6 KO
CD58 KO
CMTM6 OE
CMTM6
HSP90
HAP1
C
+CMTM6 OE
CMTM6 KO#1
CMTM6 KO#2
CMTM6
CD58
PD-L1
HSP90
8505C
D
+CMTM6 OE
+CMTM6 OE
CMTM6 KO#6
CMTM6 KO#12
IFNγ
CMTM6
CD58
PD-L1
HSP90
A375
E
+CMTM6 OE
CMTM6 KO#1
CMTM6 KO#2
CMTM6
CD58
PD-L1
HSP90
RKO
F
Ctrl.
+CMTM6 OE
Relative MFI of CD58
Relative MFI of PD-L1
WT
CMTM6KO#1
CMTM6KO#2
RKO
G
CMTM6 KO
CMTM6
HSP90
Relative MFI of CD58
WT
CMTM6 KO
BJAB
H
CMTM6 KO
CMTM6
HSP90
Relative MFI of CD58
WT
CMTM6 KO
Ramos
I
CMTM6 KO
CMTM6
HSP90
Relative MFI of CD58
WT
CMTM6 KO
OCI-AML2
J
MFI of CMTM6
MFI of CD58
WT
CMTM6 KD
DC
K
Relative MFI of CD58
WT
CMTM6 KO
shCD58-4
A375
L
Relative MFI of rhCD2
WT
CMTM6 KO
shCD58-4
A375

Figure S1. CMTM6 loss in HAP1 cells leads to reduced CD58 expression, Related to Figure 1

(A) Flow cytometry analysis of CD58 expression in wild-type (WT), CMTM6-knockout (CMTM6 KO), CMTM6-reconstituted (CMTM6 KO + OE) and CD58-knockout (CD58 KO) HAP1 cells.

(B) Western blot analysis of CMTM6 expression in wild-type (WT), CMTM6-knockout (CMTM6 KO), CMTM6-reconstituted (CMTM6 OE) and CD58-knockout (CD58 KO) HAP1 cells. HSP90 served as a control.

(C-E) Western blot analysis of CMTM6, CD58, and PD-L1 in wild-type (WT), CMTM6-knockout (CMTM6 KO), CMTM6-overexpressing (WT+CMTM6 OE), and CMTM6-reconstituted (CMTM6 KO+CMTM6 OE) 8505C cells (C), A375 cells with or without IFNγ exposure (D), and RKO cells (E). HSP90 served as a control.

(F) Flow cytometry analysis of CD58 and PD-L1 expression in wild-type (WT), CMTM6-knockout (CMTM6 KO), CMTM6-overexpressing (WT+CMTM6 OE), and CMTM6-reconstituted (CMTM6 KO+CMTM6 OE) RKO cells. Data represent the mean ± standard deviation of triplicates and were analyzed using a two-way ANOVA test (with Tukey's multiple comparisons test). A p-value greater than 0.05 indicates non-significance (ns), while a p-value less than 0.0001 is denoted as **.

(G-I) Western blot analysis of CMTM6 and flow cytometry analysis of CD58 expression in wild-type (WT) and CMTM6-knockout (CMTM6 KO) B-cell lymphoma cells – BJAB (G), Ramos (H) and acute myeloid leukemia cells - OCI-AML2 (I). HSP90 served as a control.

(J) Flow cytometry analysis of CMTM6 and CD58 expression in wild-type (WT) and CMTM6-knockdown (CMTM6 KD) primary dendritic cells that were generated from human peripheral blood-derived progenitors.

(K) Flow cytometry analysis of CD58 expression in wild-type (WT), CMTM6-knockout (CMTM6 KO), and CD58-knockdown (shCD58-4) A375 cells.

(L) Flow cytometry analysis of rhCD2 binding in wild-type (WT), CMTM6-knockout (CMTM6 KO), and CD58-knockdown (shCD58-4) A375 cells described in (K).

The data are presented as mean ± standard deviation (SD) of triplicates and were analyzed using unpaired two-tailed student's t-test (G-J) or one-way ANOVA (with Tukey's multiple comparisons test) (K-L). The statistical significance levels are indicated as follows: ns (not significant; $p \geq 0.05$), * ($p < 0.05$), and ** ($p < 0.0001$).
