# Supplementary material for: CMTM6 shapes antitumor T cell response through modulating protein expression of CD58 and PD-L1
Source: Cancer Cell. Author manuscript; Available in PMC 2024 May 23. (PMC11113010; doi:10.1016/j.ccell.2023.08.008)
Supplement: S2 [file NIHMS1981828-supplement-S2.pdf]

Figure S2

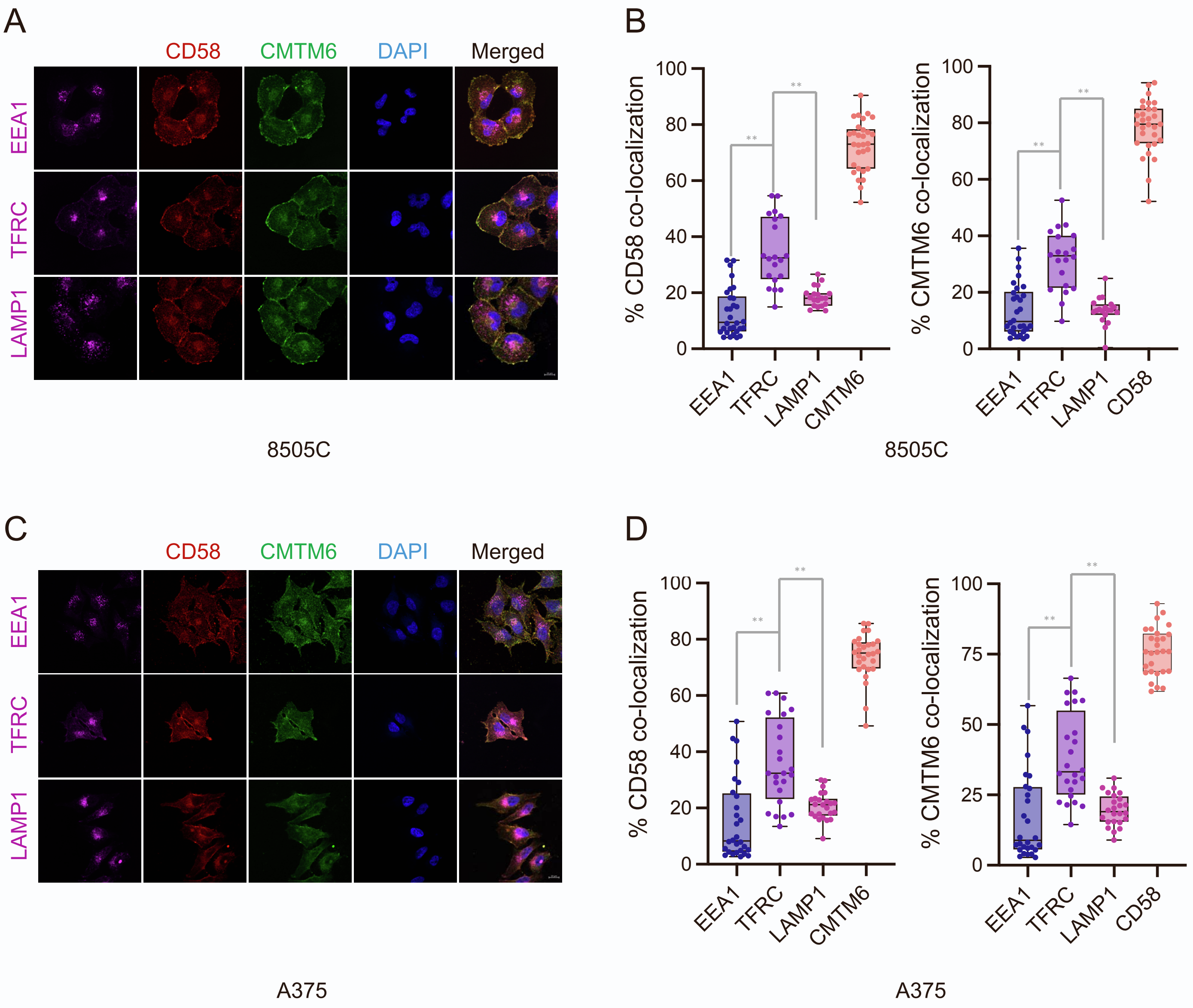
A
CD58
CMTM6
DAPI
Merged
EEA1
TFRC
LAMP1
8505C
B
% CD58 co-localization
% CMTM6 co-localization
100
80
60
40
20
0
**
EEA1
TFRC
LAMP1
CMTM6
CD58
8505C
C
A375
D
75
50
25

Figure S2. Subcellular localization of CMTM6 and CD58, Related to Figure 2

(A-D) Immunofluorescence imaging of CMTM6, CD58, EEA1 (an early endosome marker), TFRC (a recycling endosome marker), and LAMP1 (a late endosome/lysosome marker). Representative images of 8505C (A) and A375 (C) cells are shown. Quantification of the co-localization between CMTM6 and CD58 immunofluorescent signals, as well as the co-localization of CMTM6 or CD58 with EEA1, TFRC, and LAMP1 in 8505C (B) and A375 (D) cells. Data points on the graph represent the percentage of signal overlap in independent areas of interest. Data were analyzed using one-way ANOVA (Tukey's multiple comparisons test). $n \geq 19$. The statistical significance is denoted by $^{**}p < 0.0001$. Scale bar: 10 μm.
