# Supplementary material for: CMTM6 shapes antitumor T cell response through modulating protein expression of CD58 and PD-L1
Source: Cancer Cell. Author manuscript; Available in PMC 2024 May 23. (PMC11113010; doi:10.1016/j.ccell.2023.08.008)
Supplement: S3 [file NIHMS1981828-supplement-S3.pdf]

Figure S3

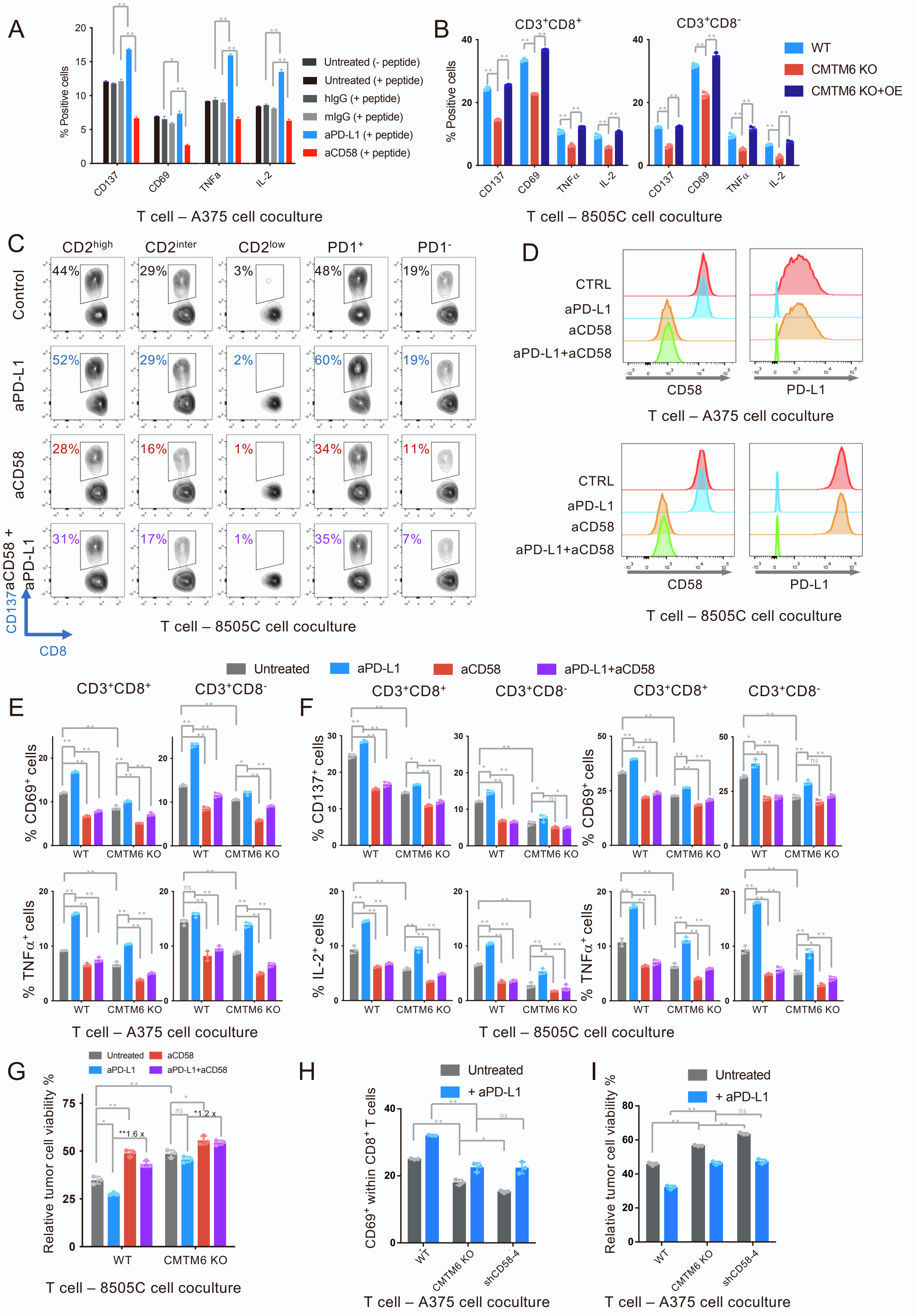
A
% Positive cells
CD137
CD69
TNFa
IL-2
Untreated (- peptide)
Untreated (+ peptide)
hIgG (+ peptide)
mIgG (+ peptide)
aPD-L1 (+ peptide)
aCD58 (+ peptide)
T cell – A375 cell coculture
B
CD3+CD8+
CD3+CD8-
% Positive cells
WT
CMTM6 KO
CMTM6 KO+OE
T cell – 8505C cell coculture
C
CD2high
CD2inter
CD2low
PD1+
PD1-
Control
aPD-L1
aCD58
aCD58 + aPD-L1
44%
29%
3%
48%
19%
52%
29%
2%
60%
19%
28%
16%
1%
34%
11%
31%
17%
1%
35%
7%
CD137
CD8
T cell – 8505C cell coculture
D
CTRL
aPD-L1
aCD58
aPD-L1+aCD58
CD58
PD-L1
T cell – A375 cell coculture
T cell – 8505C cell coculture
Untreated
aPD-L1
aCD58
aPD-L1+aCD58
E
CD3+CD8+
CD3+CD8-
% CD69+ cells
% TNFα+ cells
WT
CMTM6 KO
T cell – A375 cell coculture
F
% CD137+ cells
% CD69+ cells
% IL-2+ cells
% TNFα+ cells
T cell – 8505C cell coculture
G
Relative tumor cell viability %
**1.6 x
*1.2 x
WT
CMTM6 KO
T cell – 8505C cell coculture
H
Untreated
+ aPD-L1
CD69+ within CD8+ T cells
WT
CMTM6 KO
shCD58-4
T cell – A375 cell coculture
I
Untreated
+ aPD-L1
Relative tumor cell viability %
WT
CMTM6 KO
shCD58-4
T cell – A375 cell coculture

Figure S3. Influence of CMTM6, CD58, and PD-L1 on antigen-specific T cell – tumor cell interactions, Related to Figure 3

(A) Flow cytometry analysis of T cells in the T cell-tumor cell coculture system (illustrated in Figure 3A). A375 cells with (+ peptide) or without (- peptide) MART-1 peptide loading were cultured with MART-1 TCR transduced T cells. The cocultures of A375 cells with T cells were treated with blocking antibodies against CD58 (aCD58, isotype: mIgG) or PD-L1 (aPD-L1, isotype: hIgG), as indicated. Human normal IgG (hIgG) and mouse normal IgG (mIgG) served as controls. After 24 hours of coculture, expression of indicated T cell activation markers and cytokines in $CD3^{+}CD8^{+}$ cells was determined by flow cytometry. To allow detection of TNFα, and IL-2, brefeldin A was added 4 h before T cell harvesting.

(B) Effect of CMTM6 loss on T cell activation. MART-1 TCR transduced T cells were cocultured with MART-1 peptide-loaded CMTM6-wild-type, CMTM6-knockout (CMTM6 KO) or CMTM6-reconstituted (CMTM6KO+OE) 8505C cells, as indicated. After 24h of coculture, expression of indicated T cell activation markers and cytokines in $CD8^{+}$ and $CD8^{-}$ T cells was determined by flow cytometry. To allow detection of TNFα and IL-2, brefeldin A was added 4 h before T cell harvesting.

(C) Flow cytometry analysis of T cells that were cocultured for 18 h with 8505C cells in the absence or presence of the indicated blocking antibodies. Expression of CD137 was analyzed within $CD3^{+}CD8^{+}$ cell populations stratified by CD2 expression ($CD2^{high}$: top 33%, $CD2^{inter}$: middle 33%, $CD2^{low}$: bottom 33%), as well as PD-1 expression ($PD\text{-}1^{+}$ or $PD\text{-}1^{-}$). Representative contour plots are presented.

(D) Flow cytometry analysis of CD58 and PD-L1 staining on A375 and 8505C cells incubated with control or blocking antibodies against CD58 or PD-L1. After T cell-tumor cell coculture in the presence or absence of blocking antibodies as indicated, tumor cells were washed and stained with mixed fluorophore-conjugated anti-CD58 and anti-PD-L1 antibodies. Blocking antibodies against CD58 or PD-L1 in the coculture prevented the post-coculture staining.

(E-G) Effect of PD-L1 blockade, CD58 blockade, and CMTM6 deletion on T cell activation and tumor cell viability. MART-1 TCR transduced T cells were cocultured with MART-1 peptide-loaded tumor cells in the presence of PD-L1 blocking antibody (atezolizumab) (aPD-L1), CD58 blocking antibody (aCD58), or their combination. The coculture without antibody treatment (untreated) served as control. After 18-24h of coculture, T cell activation and tumor cell viability were analyzed.

(E-F) Flow cytometry analysis of T cells that were cocultured with CMTM6-wildtype (WT) and CMTM6-knockout (CMTM6 KO) A375 cells (E) or 8505C cells (F) in the absence or presence of the indicated blocking antibodies. Expression of CD137, CD69, IL-2, and TNFα within the $CD3^{+}CD8^{+}$ and $CD3^{+}CD8^{-}$ cell populations was depicted as indicated.

(G) Viability of CMTM6-wildtype and -knockout 8505C cells after coculture with T cells for 60 h was determined by CellTiter-Blue® Cell Viability Assay. The data presented depict relative tumor cell viability (relative to tumor cells that were cultured in the absence of T cells).

(H) Flow cytometry analysis of MART-1 TCR transduced T cells that were cocultured with MART-1 peptide-loaded CMTM6-wildtype (WT), CMTM6-knockout (CMTM6 KO), and CD58 knockdown (shCD58-4) A375 cells in the absence or presence of PD-L1 blocking antibody treatment. As Figure S1K shows, the levels of CD58 expression in CMTM6 KO and shCD58-4 A375 cells are comparable. The percentages of $CD69^+$ within $CD3^+CD8^+$ cells are shown.

(I) Viability of MART-1 peptide loaded CMTM6-wildtype, CMTM6-knockout, and CD58 knockdown (shCD58-4) cells after the coculture with MART-1 TCR transduced T cells in the absence or presence of PD-L1 blocking antibody (as described in Figure S3H) was determined by CellTiter-Blue® Cell Viability Assay**.** The data presented depict relative tumor cell viability (relative to tumor cells that were cultured in the absence of T cells).

Data represent mean ± standard deviation of at least triplicates (A-B) (E-I) and were analyzed using two-way ANOVA (Tukey's multiple comparisons test). The statistical significance levels are denoted as follows: ns (not significant; $p \geq 0.05$), * ($p < 0.05$), and ** ($p < 0.0001$).

Figure S4

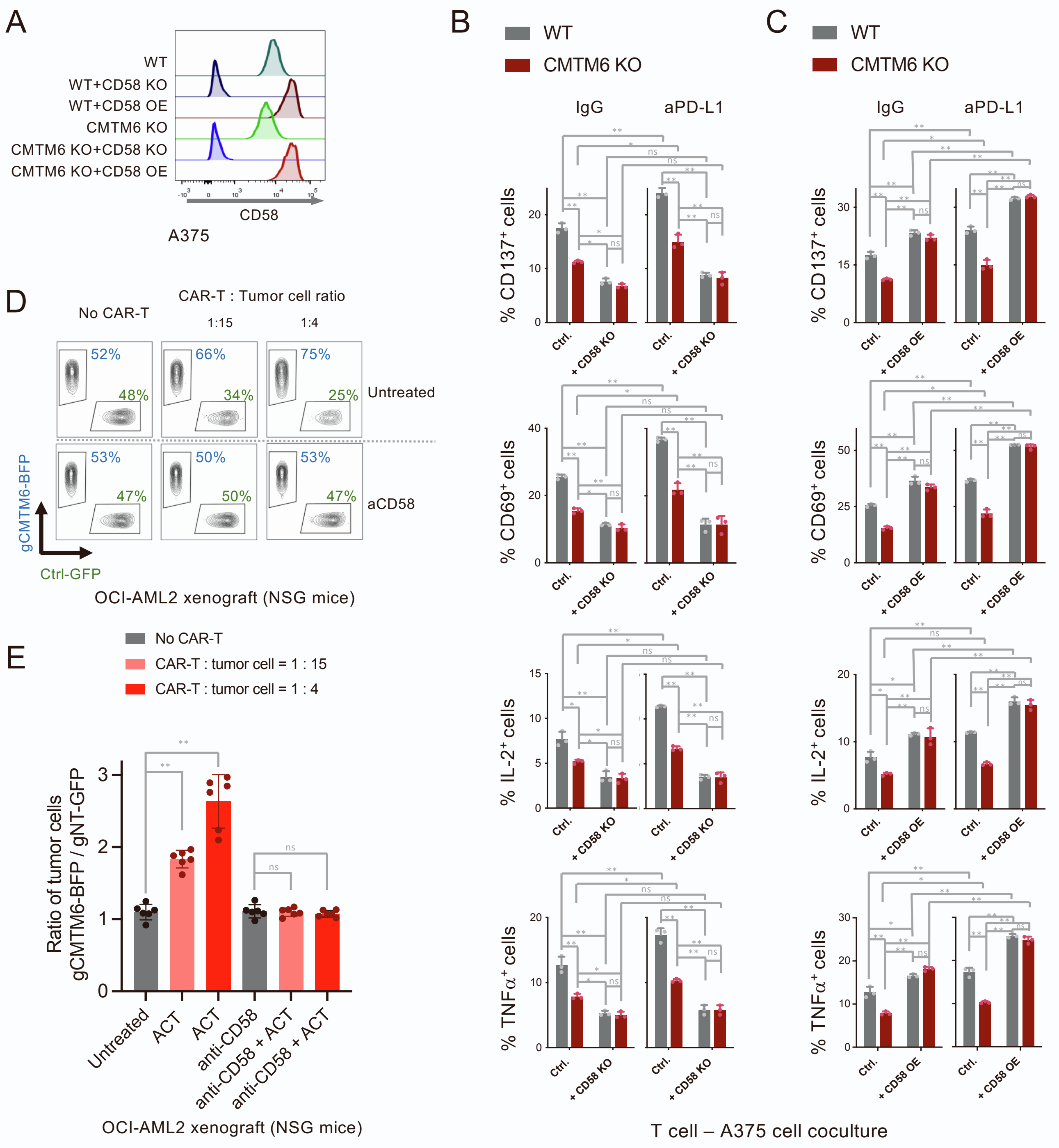
A
WT
WT+CD58 KO
WT+CD58 OE
CMTM6 KO
CMTM6 KO+CD58 KO
CMTM6 KO+CD58 OE
CD58
A375
B
WT
CMTM6 KO
IgG
aPD-L1
% CD137+ cells
% CD69+ cells
% IL-2+ cells
% TNFα+ cells
Ctrl.
+ CD58 KO
C
WT
CMTM6 KO
IgG
aPD-L1
+ CD58 OE
T cell – A375 cell coculture
D
No CAR-T
CAR-T : Tumor cell ratio
1:15
1:4
52%
48%
66%
34%
75%
25%
Untreated
53%
47%
50%
50%
53%
47%
aCD58
gCMTM6-BFP
Ctrl-GFP
OCI-AML2 xenograft (NSG mice)
E
No CAR-T
CAR-T : tumor cell = 1 : 15
CAR-T : tumor cell = 1 : 4
Ratio of tumor cells gCMTM6-BFP / gNT-GFP
Untreated
ACT
ACT
anti-CD58
anti-CD58 + ACT
anti-CD58 + ACT
OCI-AML2 xenograft (NSG mice)

Figure S4. CMTM6 and CD58 expression in tumor cells is critically involved in antitumor T cell response, Related to Figure 3

(A) Flow cytometry analysis of CD58 expression in wildtype (WT), CD58-knockout (CD58 KO), CD58-overexpression (CD58 OE), CMTM6-knockout (CMTM6 KO), CMTM6 and CD58 double-knockout (CMTM6 KO+CD58 KO), and CMTM6-knockout plus CD58-overexpression (CMTM6 KO+CD58 OE) A375 cells.

(B-C) Effects of CD58 deletion (B) or CD58 overexpression (C), in CMTM6-proficient and -deficient tumor cells on T cell activation. MART-1 TCR transduced T cells were cocultured with A375 cells loaded with MART-1 peptide and carrying genetic modifications as indicated. The cocultures were conducted in the presence or absence of a PD-L1 blocking antibody, with normal human IgG serving as a control. Expression of $CD137^+$, $CD69^+$, IL-2^+, and $TNF\alpha^+$ cells within the $CD3^+CD8^+$ cell population was determined by flow cytometry.

(D-E) Effects of CMTM6 and CD58 on response to CAR-T treatment in an *in vivo* xenograft model of acute myeloid leukemia (AML). CMTM6-proficient and -deficient OCI-AML2 cells were individually labeled with GFP (gNT-GFP) and BFP (gCMTM6-BFP), respectively, and transplanted into NSG mice at a 1:1 ratio. The coding sequences of GFP and BFP fluorescent proteins were separately integrated into constructs containing different gRNAs: a non-targeting control gRNA for GFP and a CMTM6-targeting gRNA for BFP. Following treatment with anti-CD33 CAR-T cells at the specified CAR-T cell:tumor cell ratio for seven hours, peripheral blood was collected, and the ratio of GFP and BFP-positive AML cells was analyzed using flow cytometry. In the CD58 blockade group, an anti-CD58 antibody was administered 12 hours before the injection of AML cells.

(D) Flow cytometry analysis was performed to examine the presence of GFP (gNT) and BFP (gCMTM6)-positive AML cells remaining in the peripheral blood of the mice after CD33 CAR-T treatment, both in the presence and absence of CD58 blockade. The untreated group served as the control. Representative contour plots were generated.

(E) The ratios of BFP (gCMTM6)-positive/GFP (gNT)-positive AML cells, as described in (D), were quantified.

Data represent mean ± standard deviation of replicates (B-C n=3; E n=6) and were analyzed using two-way (B-C) or one-way (E) ANOVA (Tukey's multiple comparisons test). The statistical significance levels are indicated as follows: ns (not significant; $p \geq 0.05$), * ($p < 0.05$), and ** ($p < 0.0001$).
