# Supplementary material for: CMTM6 shapes antitumor T cell response through modulating protein expression of CD58 and PD-L1
Source: Cancer Cell. Author manuscript; Available in PMC 2024 May 23. (PMC11113010; doi:10.1016/j.ccell.2023.08.008)
Supplement: S4 [file NIHMS1981828-supplement-S4.pdf]

Figure S4

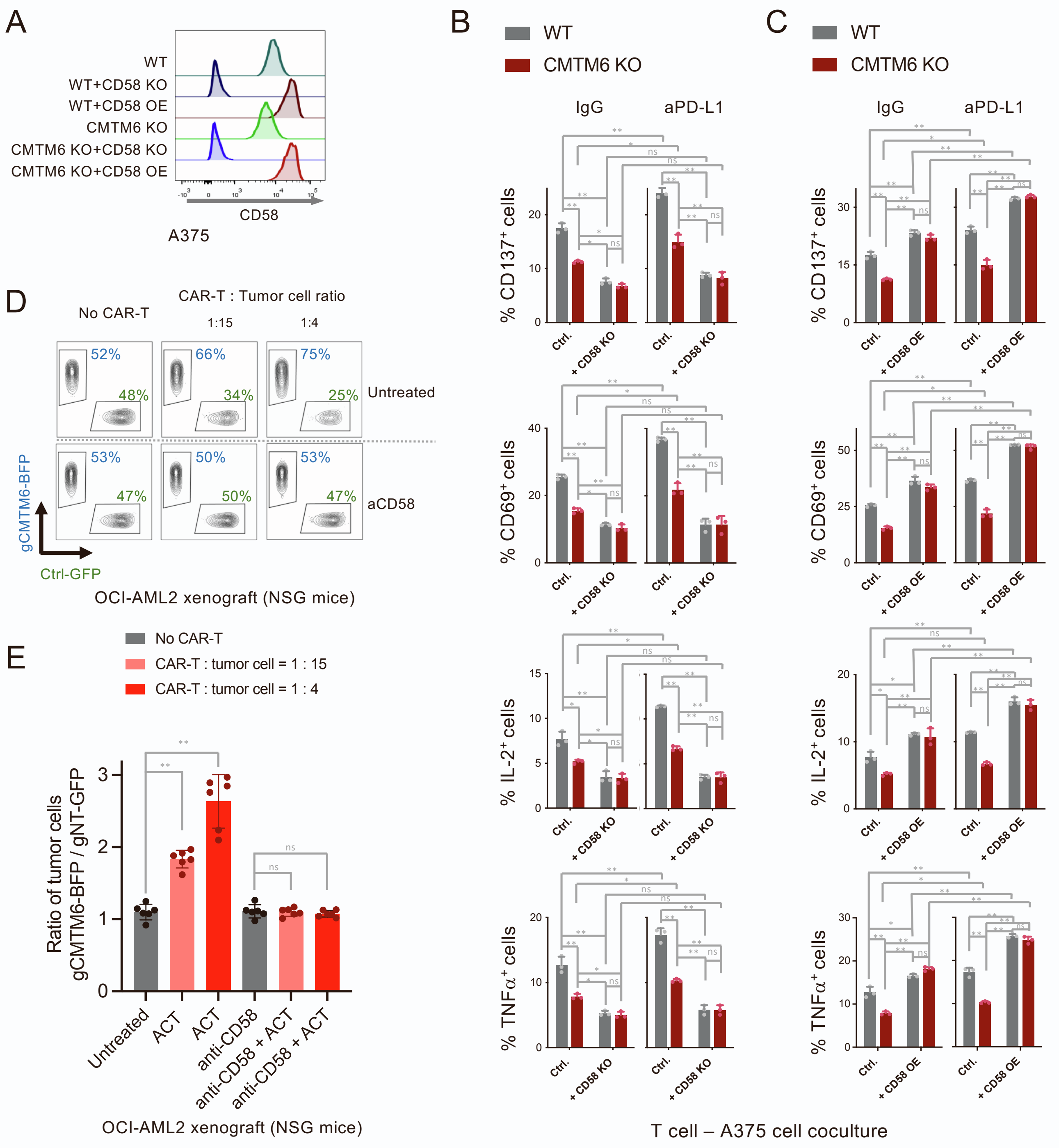

A
WT
WT+CD58 KO
WT+CD58 OE
CMTM6 KO
CMTM6 KO+CD58 KO
CMTM6 KO+CD58 OE
CD58
A375
B
WT
CMTM6 KO
IgG
aPD-L1
% CD137+ cells
% CD69+ cells
% IL-2+ cells
% TNFα+ cells
Ctrl.
+ CD58 KO
C
WT
CMTM6 KO
IgG
aPD-L1
+ CD58 OE
T cell – A375 cell coculture
D
No CAR-T
CAR-T : Tumor cell ratio
1:15
1:4
52%
48%
66%
34%
75%
25%
Untreated
53%
47%
50%
50%
53%
47%
aCD58
gCMTM6-BFP
Ctrl-GFP
OCI-AML2 xenograft (NSG mice)
E
No CAR-T
CAR-T : tumor cell = 1 : 15
CAR-T : tumor cell = 1 : 4
Ratio of tumor cells
gCMTM6-BFP / gNT-GFP
Untreated
ACT
ACT
anti-CD58
anti-CD58 + ACT
anti-CD58 + ACT
OCI-AML2 xenograft (NSG mice)

Figure S4. CMTM6 and CD58 expression in tumor cells is critically involved in antitumor T cell response, Related to Figure 3

(A) Flow cytometry analysis of CD58 expression in wildtype (WT), CD58-knockout (CD58 KO), CD58-overexpression (CD58 OE), CMTM6-knockout (CMTM6 KO), CMTM6 and CD58 double-knockout (CMTM6 KO+CD58 KO), and CMTM6-knockout plus CD58-overexpression (CMTM6 KO+CD58 OE) A375 cells.

(B-C) Effects of CD58 deletion (B) or CD58 overexpression (C), in CMTM6-proficient and -deficient tumor cells on T cell activation. MART-1 TCR transduced T cells were cocultured with A375 cells loaded with MART-1 peptide and carrying genetic modifications as indicated. The cocultures were conducted in the presence or absence of a PD-L1 blocking antibody, with normal human IgG serving as a control. Expression of $CD137^+$, $CD69^+$, $IL\text{-}2^+$, and $TNF\alpha^+$ cells within the $CD3^+CD8^+$ cell population was determined by flow cytometry.

(D-E) Effects of CMTM6 and CD58 on response to CAR-T treatment in an *in vivo* xenograft model of acute myeloid leukemia (AML). CMTM6-proficient and -deficient OCI-AML2 cells were individually labeled with GFP (gNT-GFP) and BFP (gCMTM6-BFP), respectively, and transplanted into NSG mice at a 1:1 ratio. The coding sequences of GFP and BFP fluorescent proteins were separately integrated into constructs containing different gRNAs: a non-targeting control gRNA for GFP and a CMTM6-targeting gRNA for BFP. Following treatment with anti-CD33 CAR-T cells at the specified CAR-T cell:tumor cell ratio for seven hours, peripheral blood was collected, and the ratio of GFP and BFP-positive AML cells was analyzed using flow cytometry. In the CD58 blockade group, an anti-CD58 antibody was administered 12 hours before the injection of AML cells.

(D) Flow cytometry analysis was performed to examine the presence of GFP (gNT) and BFP (gCMTM6)-positive AML cells remaining in the peripheral blood of the mice after CD33 CAR-T treatment, both in the presence and absence of CD58 blockade. The untreated group served as the control. Representative contour plots were generated.

(E) The ratios of BFP (gCMTM6)-positive/GFP (gNT)-positive AML cells, as described in (D), were quantified.

Data represent mean ± standard deviation of replicates (B-C n=3; E n=6) and were analyzed using two-way (B-C) or one-way (E) ANOVA (Tukey's multiple comparisons test). The statistical significance levels are indicated as follows: ns (not significant; $p \geq 0.05$), * ($p < 0.05$), and ** ($p < 0.0001$).
