# Supplementary material for: CMTM6 shapes antitumor T cell response through modulating protein expression of CD58 and PD-L1
Source: Cancer Cell. Author manuscript; Available in PMC 2024 May 23. (PMC11113010; doi:10.1016/j.ccell.2023.08.008)
Supplement: S5 [file NIHMS1981828-supplement-S5.pdf]

Figure S5

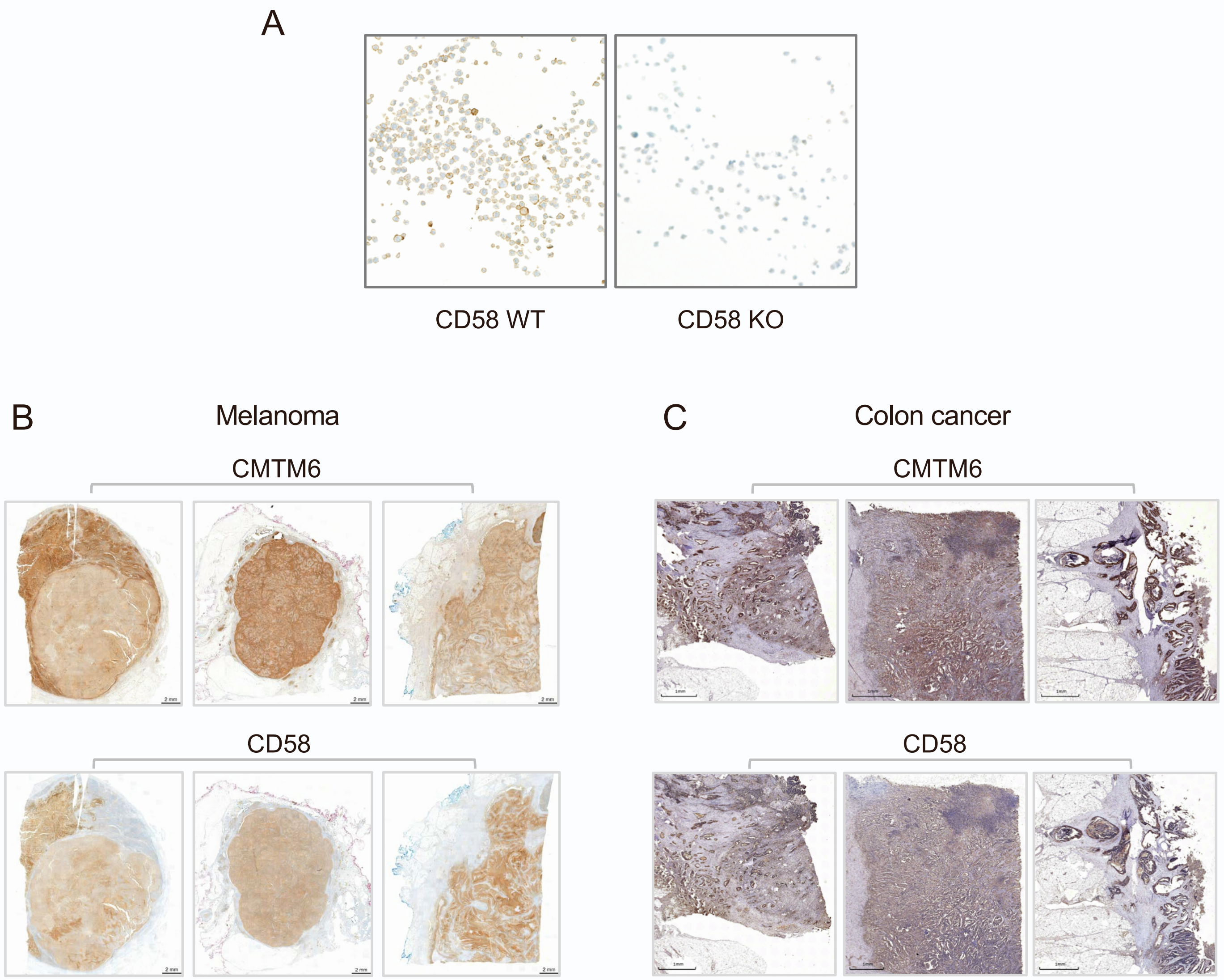
A
CD58 WT
CD58 KO
B
Melanoma
CMTM6
CD58
C
Colon cancer
CMTM6
CD58

Figure S5. Co-localization of CMTM6 and CD58 in human tumors, Related to Figure 4

(A) IHC analysis of CD58 expression in WT and CD58 KO cells. Parental (WT) and CD58-knockout (CD58 KO) 8505C cells were fixed, formalin embedded, sectioned and loaded on slides. The prepared samples were stained with a monoclonal antibody (clone #126, Novus Biologicals) for CD58 (brown). The IHC analysis of the WT and CD58 KO cells demonstrates a high degree of specificity of the CD58 antibody.

(B-C) Sequential sections from tumor biopsies of three melanoma patients (B) and three colon cancer patients (C) were stained for CMTM6 and CD58, showing co-localization of CD58 with CMTM6. Scale bar: 2mm (B); 1mm (C).
