# Supplementary material for: CMTM6 shapes antitumor T cell response through modulating protein expression of CD58 and PD-L1
Source: Cancer Cell. Author manuscript; Available in PMC 2024 May 23. (PMC11113010; doi:10.1016/j.ccell.2023.08.008)
Supplement: Table S1 [file NIHMS1981828-supplement-Table_S1.pdf]

Table S1. Patient-characteristics of the melanoma cohort, Related to Figure 4

	Overall (*n*=88)
Male (%)	54 (61.4)
Age[a] (years, median, IQR)	62.5 (53.8, 69.5)
M-stage[a,b] (%)	
M1a	16 (18.2)
M1b	8 (9.1)
M1c	44 (50.0)
M1d	20 (22.7)
BRAF V600E/K positive (%)	31 (35.2)
Lactate dehydrogenase[a] (median, IQR)	189.0 (156.0, 238.0)
Treatment before biopsy (%)	
Ipilimumab	43 (48.9)
Targeted therapy	14 (15.9)
TIL-therapy	1 (1.1)
Treatment (%)	
Anti-PD1 monotherapy	82 (93.2)
Anti-PD1 + anti-CTLA4	6 (6.7)

[a]At start treatment; [b] M-stage according to the 8th edition American Joint Committee on Cancer (AJCC8) cancer staging system. IQR, interquartile range.
